# Systematic Review of the Links between Eco-Distress and Mental Health

**DOI:** 10.1007/s10393-025-01769-z

**Published:** 2025-12-02

**Authors:** N. Gebhardt, F. Westermann, Lilli Kleinböhl, H.-C. Friederich, C. Nikendei

**Affiliations:** 1 Department of General Internal Medicine and Psychosomatics, Heidelberg University Hospital, Heidelberg, Germany; 2 University of Innsbruck, Innsbruck, Austria

**Keywords:** eco-anxiety, climate anxiety, mental health impairments, psychometric quality, psychological assessment

## Abstract

**Background:**

Eco-distress shows positive correlations with mental health impairments. However, the correlations reported by different studies vary widely. The aim of our review was to explore possible methodological sources for this heterogeneity, hereby deepening our understanding of eco-distress and its relationship with mental health.

**Methods:**

Following PRISMA guidelines, Academic Search Complete, PubMed, PsycInfo, and Web of Science were searched on 15.03.2025 for publications reporting the initial development of instruments assessing eco-distress, subsequent validation studies, or correlations with mental health impairments. We analyzed the relationship of psychometric quality and the range of effect sizes descriptively, as well as the potential influence of the type of mental health questionnaire, the psychometric quality, and study and sample characteristics statistically as moderators in meta-analysis.

**Results:**

We included 87 studies reporting on 15 different instruments. The underlying definitions of eco-distress differed and psychometric quality was mixed. However, confidence intervals did not vary systematically due to psychometric quality. Overall, eco-distress and mental health impairments correlated with *r* = 0.32, 95% CI [0.27; 0.37]. Only type of mental health questionnaire moderated the effect size.

**Conclusion:**

Although it varied in magnitude, there was a significant positive correlation between eco-distress and mental health impairments. However, type of eco-distress questionnaire did not systematically influence the effect size. The heterogeneity was considerable and could only be partially explained by the moderators used. Future research should focus on the psychometric evaluation of existing instruments, as information on psychometric quality was incomplete for many instruments.

**Supplementary Information:**

The online version contains supplementary material available at 10.1007/s10393-025-01769-z.

## Background

Climate change, in conjunction with other environmental crises, poses a serious threat to human livelihood (IPCC, 2023). Awareness of this threat can trigger negative emotions such as anxiety, sadness, anger, or guilt (Pihkala, 2022), often subsumed under terms like climate anxiety, eco-anxiety, or eco-distress. While most research agrees that anxiety related to anthropogenic climate change is at the core of the concept (Wullenkord et al., 2025), no standard definition or standard term exists (Boivin et al., 2025; Coffey et al., 2021). Since the concept encompasses more emotions than just anxiety, this review uses the term eco-distress. While other concepts of emotional reactions towards climate change exist, such as eco-grief or eco-guilt (Ágoston et al., 2022a, b), eco distress has been studied most extensively and is the construct encompassing more aspects than just the emotional response. Specifically, eco-distress may include cognitive indicators, such as difficulties concentrating, physiological indicators, such as muscle tension, and behavioral indicators, such as social withdrawal (van Valkengoed et al., 2023). As these indicators show high similarities with definitions of mental health impairments, eco-distress is of particular interest to mental health research. In severe cases, eco-distress might result in functional impairments (Ágoston et al., 2022a, b), and it has been shown to correlate with syndromes of general distress, anxiety, or depression (Boluda-Verdú et al., 2022; Gago et al., 2024). Thus, high levels of eco-distress are of relevance to mental health care and warrant professional evaluation and psychosocial support (Cianconi et al., 2023; Clayton et al., 2017).

Understanding the interplay between eco-distress and other mental health impairments is crucial for the development of appropriate prevention and treatment measures. However, the exact nature of the relationship between eco-distress and mental impairments remains unclear, and there is a large heterogeneity in the correlations of eco-distress with different syndromes of mental health impairments (Cosh et al., 2024). This could be caused by various methodological and construct-related reasons. For one, using instruments of poor psychometric quality can result in measurement inaccuracy. If this was the case, correlations should be consistent for instruments with good psychometric quality and show wider ranges for instruments with poor psychometric quality. Secondly, different instruments could assess different concepts. If this was the case, correlations of eco-distress with mental health impairments would vary between instruments, but would be relatively consistent per instrument. Indeed, a recent review compared twelve eco-distress questionnaires and identified 57 disparate indicators assessed by the various instruments, pointing towards heterogeneity in the underlying concepts of eco-distress (van Dijk et al., 2025). Thirdly, sample and study characteristics such as age or gender could have an influence on the size of the correlation. For example, women show higher values of both eco-distress and depression (Gebhardt et al., 2023; Kocalevent et al., 2013; Rief et al., 2004). Thus, it seems plausible that gender might moderate the correlation of eco-distress and mental health. Finally, item overlap with instruments assessing mental health impairments could have an influence on the size of the correlation. Item overlap describes the extent to which the items of two different questionnaires are asking for the same set of indicators. If item overlap explained the variability of the correlation of eco-distress and mental health impairments, eco-distress questionnaires sharing many items with syndromes of mental health impairments should show higher correlations with the respective syndromes.

Recently, a number of reviews have either summarized the content of eco-distress questionnaires and assessed their psychometric quality (Owczarek et al., 2025; Ramsay et al., 2025) or summarized their correlations with mental health impairments (Cosh et al., 2024; Gago et al., 2024; Pitt et al., 2023). However, none have combined these two aspects to explore possible sources of heterogeneity in correlations of eco-distress and mental health impairments. Our aim was to close this gap by systematically comparing the size of correlations of eco-distress and mental health impairments depending on i) the psychometric quality of the eco-distress questionnaire; ii) the underlying concept of eco-distress; iii) sample and study characteristics; and iv) the extent of item overlap with an anxiety disorder or a depressive disorder. To this end, we conducted a systematic review of the available eco-distress questionnaires and included studies that reported either on the psychometric quality of the questionnaires, the correlation with mental health impairments, or both.

## Methods

### Information Sources, Search Strategy and Eligibility Criteria

**Figure 1:**
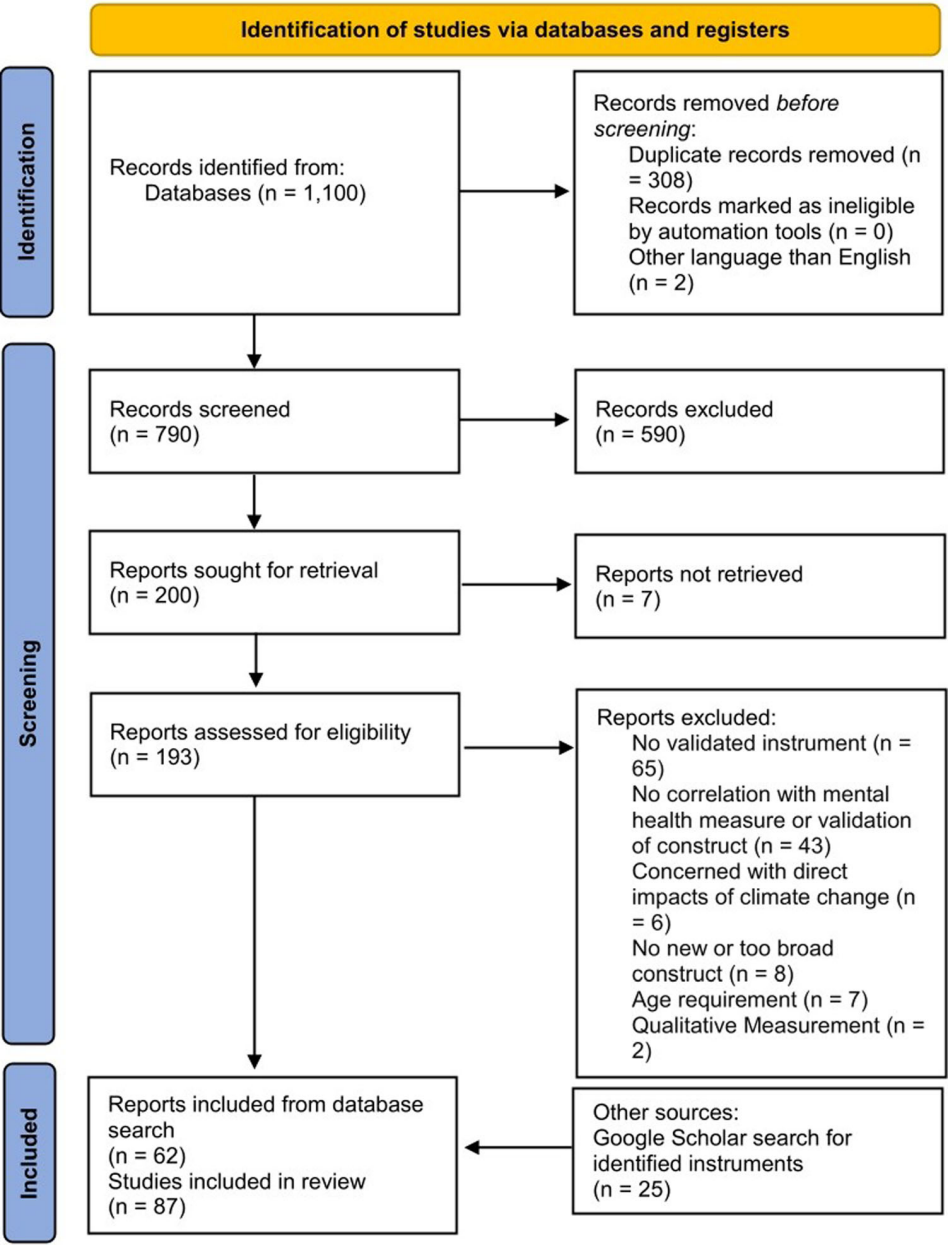
Flow chart of the selection process of studies to be included in the scoping review.

This review adhered to the Preferred Reporting Items for Systematic reviews and Meta-Analyses (PRISMA) (Page et al., 2021). In a first step, a literature search was conducted on 15.03.2025 on Academic Search Complete, PubMed, PsycInfo, and Web of Science. We did not exclude studies based on publication year. The search term consisted of different expressions referring to climate change-related distress, for example “eco-anxiety” or “eco-distress” and expressions for self-report questionnaires, for example “scale” or “questionnaire”. The full search term is provided as Online Appendix A. The expressions were generated through forward reference screening of studies included in reviews on eco-distress and mental health. Studies were deemed eligible if they were published in English or German in a peer-reviewed journal and presented original, quantitative data on the validation of an instrument assessing eco-distress, or on associations of instruments assessing eco-distress with mental health impairments. Studies on individuals younger than 18 years were excluded. We took this step to enhance the comparability of the studies included in our review, as youth in different stages of psychosocial development might understand the same eco-distress questionnaire in a qualitatively different way. For instance, the PHQ-9 as a valid measure of depression in adult samples has been shown to result in more false positives in a sample of children aged 10–12, and the cut-off values were considerably lower than in the general population (Stewart et al., 2023). In a second step, the names of all instruments extracted from the literature search were entered as exact search terms into Google Scholar, and all results were screened for additional publications which met the inclusion criteria. The full process is presented as a flow chart in Fig. 1.

### Selection of Results

The screening of the resulting literature was performed by F.W., L.K., and N.G. Each record was assessed independently by two researchers. Publications which had not been included by both researchers were discussed until consensus was reached. Thereafter, information was extracted regarding all objectives of the review by one researcher and checked by at least one other team member.

### Charting of Results

*Concepts of eco-distress*

To evaluate the underlying concepts of eco-distress, we extracted the definition of eco-distress the instrument was based on from the original publication. Moreover, we extracted the number of factors and items per factor and whether the items included emotional, cognitive, behavioral, and/or physiological indicators. This conceptualization is well-established in emotion research and was thus deemed helpful to compare the different instruments in a systematic way (van Valkengoed et al., 2023).

*Correlations of Eco-Distress and Mental Health*

We extracted all correlations of eco-distress questionnaires with questionnaires assessing mental health impairments.

*Sample and study characteristics*

From all studies reporting correlations of eco-distress questionnaires and mental health, we extracted age, gender, region, mental health questionnaire, and item overlap as potential moderators of the correlation of eco-distress with mental health. We grouped the questionnaires assessing mental health impairments into four categories, namely anxiety, depression, stress, and well-being. We chose these four categories bottom-up due to data availability: while there was sufficient data on correlations with these four constructs, the same did not hold for constructs such as posttraumatic stress or mindfulness. To evaluate item overlap with mental health impairments, we decided to focus on overlap with symptoms of depression and anxiety because the majority of studies assessing correlations of eco-distress with mental health impairments employs mental health questionnaires which assess depression and/or anxiety (Pitt et al., 2023). For each eco-distress questionnaire, we compared item content with the definition of a generalized anxiety disorder or a depressive disorder according to the International Classification of Diseases, 11th revision [ICD-11, World Health Organization (WHO), 2019]. We rated item overlap dichotomous as either “yes” or “no” for each item. Herein, we focused solely on the emotion or behavior named by the item, regardless of its context. For example, anxiety due to the climate crisis was rated as a “yes” for item overlap with a generalized anxiety disorder and as a “no” for item overlap with a depressive disorder. As some symptoms are part of the diagnostic criteria for both disorders (for example sleep disturbances), these were rated “yes” for item overlap with both disorders. Item overlap was expressed as a percentage. The percentages are reported as part of Online Appendix D.

### Analysis

*Psychometric quality of the eco-distress questionnaires.* To evaluate the psychometric quality of the instruments, we followed the recommendations of the COSMIN guideline for systematic reviews of patient-reported outcome measures, version 2.0 (Mokkink et al., 2024). In a first step, we assessed the risk of bias (RoB) for the original publications of each instrument. In a second step, we assessed the psychometric quality of the instrument. In a third step, we extracted common indicators of psychometric quality from additional studies employing the respective questionnaire and synthesized these into one overall rating. This rating was descriptively compared against the variations in the range of the correlations of the respective questionnaires with mental health impairments.

*Influence of the underlying concept of eco-distress, sample and study characteristics, item overlap.* To analyze the potential influence of the methodological and construct-related aspects, we ran multivariate random-effects meta-analyses with different moderators. Calculations were based on the metafor package in R (Viechtbauer, 2010). We chose a hierarchical model with random effects per study to account for the non-independence of observations which were drawn from the same sample. Category of mental health questionnaire, eco-distress questionnaire, mean age of participants, percentage of female participants, region of study, and percentage of item overlap were entered as potential moderators. We retained a moderator if it improved the model fit (likelihood ratio test) and explained a significant share of the variance (pseudo *R*^*2*^). Item overlap as a moderator was only tested for the subset of mental health questionnaires assessing the respective syndrome. That is, the percentage of item overlap with the construct of anxiety was only tested for correlations of eco-distress questionnaires and anxiety questionnaires, and the percentage of item overlap with the construct of depression was only tested for correlations of eco-distress questionnaires and depression questionnaires. If correlations were reported per subscale of an eco-distress questionnaire, we entered them separately. To address the issue that correlation coefficients can be biased in case of sample heterogeneity (Hassler & Thadewald, 2003), we transformed the correlation coefficients to Fisher’s z-values. For better interpretability, all results reported in this manuscript were transformed back into Pearsons’s *r* correlation coefficients.

## Results

### Literature search

The literature search yielded 1,100 results. After removal of duplicates and studies in another language than English or German, 790 abstracts were screened, and 200 studies were selected for full-text analysis. Out of these, 7 could not be retrieved, thus 193 studies were assessed for eligibility. Of those, 62 met the inclusion criteria and were included in the final review. The search of Google Scholar added 25 more studies to our review. Hence, 87 studies were included in our review. The flow chart of the article selection is provided in Fig. 1.

### Underlying Concepts of Eco-Distress

In total, 15 instruments were retrieved from the 87 studies that proved eligible. Of these, all instruments assessed emotional indicators of eco-distress, while the inclusion of items assessing cognitive, behavioral, or physiological indicators varied. Emotional indicators assessed were different forms of anxiety (worry, fear, panic) and facets of sadness and anger. One instrument (Climate-Anxiety-Climate-Hope-Index (Sangervo et al., 2022)) asked for hope and therefore for an item with positive valence. Cognitive indicators were assessed by ten of the instruments, most often by items asking for difficulties concentrating or recurrent thoughts about climate change or environmental degradation. Behavioral indicators were assessed by eight of the instruments, most often asking for sleep disturbances and impairments in social interactions. Physiological indicators were assessed by seven of the instruments, most often asking for muscle tension and nausea. Five of the instruments, the Climate Change Anxiety Scale (Clayton & Karazsia, 2020), the Climate Change Distress and Impairment Scale (Hepp et al., 2023), the Eco Anxiety Questionnaire (Ágoston et al., 2022a, b), the EMEA Eco Anxiety Scale (Jalin et al., 2025), and the Hogg Eco-Anxiety Scale (T. L. Hogg et al., 2021), included items assessing all four indicators. An overview is given in Table 1.

**Table 1:** Definitions of eco-distress and indicators assessed for all instruments included in the review

Questionnaire	Scale design	Concept of eco-distress	Indicators assessed			
			Cog	Beh	Emo	Phy
Anticipatory solastalgia Scale	Unimodal (*i* = 5)	Multifaceted emotional response, reflects present distress stemming from concerns about future environmental degradation			x	
Brief solastalgia scale	Unimodal (*i* = 5)	Psychological impact of environmental degradation and the loss of a once familiar environment, characterized by worry, loss, and powerlessness			x	
Climate-anxiety-climate-hope-index	*S1: Climate Anxiety Index (i* = *2);* *S2: Climate Hope Index (i* = *2)*	Mild to severe manifestations of anxiety that relate to the climate crisis and are characterized by feelings of tension, worried thoughts and physiological changes		x	x	x
Climate change anxiety scale	*S1: Cognitive & emotional* *impairment (i* = *8);* *S2: Functional impairment (i* = *5*)	Clinically significant anxious response to climate change, which is distinct from general worry and can result in impairments on the cognitive and functional level	x	x	x	x
Climate change distress and impairment scale	*S1: Distress (i* = *15);* *S2: Impairment (i* = *8)*	Non-pathological affective response to climate change, encompassing anxiety, anger, and sadness	x	x	x	x
Climate change distress scale	*S1: Climate change anxiety (i* = *?);* *S2: Climate change hopelessness* *(i* = *?)*	Heightened level of concern about climate change that leads to negative emotional responses, including anxiety, depression, stress, and a sense of helplessness, which may interfere with daily life			x	
Climate change version – man-made disaster-related distress scale	*S1: Psychological distress (i* = *11)* *S2: Change of existing belief systems (i* = *5)*	Psychological distress, like anxiety, anger, worry and doubts associated with climate change and the awareness of its anthropogenic origin, accompanied by a change in belief systems	x		x	
Climate change worry scale	Unimodal (*i* = 10)	Mainly verbal-linguistic thoughts about potential climate change scenarios and their consequences, focusing on proximal worries rather than abstract global ones	x		x	
Eco anxiety questionnaire	*S1: Habitual Ecological Worry (i* = *13);* *S2: Negative Consequences of* *Eco-Anxiety (i* = *9)*	Persistent worry about environmental degradation characterized by strong emotional reactions and varying levels of functional impairment, physical symptoms as well as feelings of helplessness	x	x	x	x
ECO-ANS-LATAM	Unimodal (*i* = 4)	Specific form of anxiety characterized by concerns about environmental and climate change, manifesting through psychological and physiological symptoms that impact an individual’s ability to function in daily life	x	x	x	
Environmental distress scale	*S1: Frequency (i* = *10);* *S2: Observation; (i* = *9);* *S3: Threat (i* = *18);* *S4: Impact (i* = *21);* *S5: Solastalgia (i* = *9*); *S6: Actions (i* = *14)*	Bio-psycho-social impact of environmental degradation and the loss of a once familiar environment, changing perception of environmental hazards, threat assessment, emotional and physical stress, as well as environmental actions	x	x	x	
EMEA eco anxiety scale	*S1: Anxiety depressive Manifestations (i* = *9)* *S2: Relational disturbances (i* = *7)* *S3: Obsession with ecology (i* = *6)*	Complex psychological phenomenon with an individual set of emotional, cognitive and relational disturbances in response to the perceived threat of climate collapse, creating potential for functional impairment	x	x	x	x
General-anxiety-disorder-climate version	Unimodal (*i* = 7)	Impact of the awareness of climate change on psychological suffering, specifically the mental health outcome generalized anxiety	x		x	x
Hogg eco-anxiety scale	*S1: Affective symptoms (i* = *4);* *S2: Rumination (i* = *3);* *S3: Behavioral symptoms (i* = *3);* *S4: Personal impact anxiety (i* = *3)*	Multidimensional psychological distress individuals experience in response to the threat of climate change and other environmental issues and their contribution to it, distinct from anxiety, depression and stress	x	x	x	x
Scale of solastalgia	*S1: Solace (i* = *7);* *S2: Algia (i* = *3)*	Psychological distress experienced by individuals in degraded settings, characterized by emotional pain and the loss of comfort associated with the familiar environment			x	

*Cog* = *Cognitive Indicators, Beh* = *Behavioral Indicators, Emo* = *Emotional Indicators, Phy* = *Physiological Indicators*

### Psychometric Quality of the Instruments

Table 2 gives a detailed overview of the studies included in our psychometric evaluation, while Table 3 offers an aggregated evaluation based on all studies included in our review that reported on the instrument, according to the COSMIN guideline (Mokkink et al., 2024). However, we did not include criterion validity and responsiveness because no standard criterion against which one could compare the instruments exists. For the Climate Change Anxiety Scale (Clayton & Karazsia, 2020), the Hogg Eco-Anxiety Scale (T. L. Hogg et al., 2021), and the Climate Change Worry Scale (Stewart, 2021), some studies reported acceptable measurement properties while others did not. In these cases, we set a “ ~ ” for the respective measurement property to distinguish them from cases in which information was not reported (“?”).The Risk of Bias Assessment for the original publications is provided as Online Appendix B. The evaluation of the psychometric quality based solely on the original publications is provided as Online Appendix C.

**Table 2:** Overview of the instruments assessing eco-distress per instrument and language, reporting study population, sampling strategy, number of participants, internal consistency, and factorial validity

Languages	Study population	Sampling Strategy	N in sample	α					Factorial Validity		
				Overall	S1	S2	S3	**S4 **	RMSEA	CFI	TLI
**Anticipatory Solastalgia Scale (ASS, *i *=5), ***unimodal scale*											
English	General pop., UK & US	Convenience	n1 = 509(Stanley, 2023)	0.91	/	/	/	/	0.08	0.99	0.98
			n2 = 493(Stanley, 2023)	0.95	/	/	/	/	0.07	0.99	0.99
	General pop., AU & NZ	Quota sampling	n1 = 1450(Stanley et al., 2025)	0.91	/	/	/	/	0.03	1.00	1.00
			n2 = 1022(Stanley et al., 2025)	0.89	/	/	/	/	0.03	1.00	1.00
**Brief Solastalgia Scale (BSS, *i *= 5), ***unimodal scale*											
English	General pop., Australia	Convenience	n = 921(Christensen et al., 2024)	0.88	/	/	/	/	0.00	1.00	/
	General pop., Australia	Conveni ence	n = 1651(Christe nsen et al., 2024)	0.89	/	/	/	/	0.02	0.99	/
	General pop., Australia	Bush fire affected	n = 802(Christensen et al., 2024)	0.91	/	/	/	/	0.03	0.99	/
**Climate-Anxiety-Climate-Hope-Index (CACHI, *i *= 4), ***S1: Climate Anxiety Index (i = 2); S2: Climate Hope Index (i = 2)*											
Finnish	General pop., FIN	Convenience	n = 2070(Sangervo et al., 2022)	/	0.77	0.69	/	/	/	/	/
**Climate Change Anxiety Scale (CCAS, *i *= 13), ***S1: cognitive & emotional impairment (i = 8); S2: functional impairment (i = 5*)											
Arabic	General pop., Egypt	Convenience	n1 = 1300(Hussein et al., 2023)	/	/	/	/	/	/	/	/
	General pop.	Convenience	n2 = 350(Abdu et al., 2024)	0.925	0.889	0.89	/	/	0.06	0.96	0.94
	General pop., LBN	Conveni ence	n3 = 763(Fekih-Romdhane et al., 2024)	0.96	0.91	0.93	/	/	0.131	0.91	/
Bahasa Indonesia	Age 18 - 35	Convenience	n = 306(Jaro’ah & Saffana, 2023)	0.91	0.87	0.83	/	/	0.091	0.98	/
Chinese	General pop.	Stratified: gender & age	n1 = 1000(Tam et al., 2023)	/	0.93	0.91	/	/	0.084	0.954	/
	General pop.^a^	Conveni ence	n2 = 1567(Lau et al., 2025)	0.915	0.840	0.83	0.859	/	0.083	0.95	0.93
	General pop.	Stratified: gender & age	n3 = 1009(Chan et al., 2024)	0.93	/	/	/	/	0.069	0.955	0.945
English	General pop., USA	Convenience	n1 = 197(Clayton & Karazsia, 2020)	/	0.97	0.94	/	/	0.070	0.93	0.92
	General pop., USA	Conveni ence	n2 =513(Cruz & High, 2022)	/	/	/	/	/	/	0.89	/
	General pop., NZ & AU	Conveni ence	n3 =401(Feather & Williams, 2022)	0.89	0.87	0.78	/	/	0.060	0.95	0.94
	General pop., AU	Convenience	n4 = 528(T. L. Hogg et al., 2023)	/	0.85	0.83	/	/	0.100	0.91	0.89
	Adolescents, PH	Convenience	n5 = 452(Simon et al., 2022)	/	/	/	/	/	0.112	0.895	0.872
	General pop., USA	Stratifie d:	n7 = 1000(Tam et al., 2023)	/	0.95	0.94	/	/	0.096	0.957	/
	Univ. students,	gender & age	n8 = 1000(Tam et al., 2023)	/	0.88	0.87	/	/	0.087	0.931	/
	General pop., IN	Convenience	n9 = 308(Stewart et al., 2023)	/	0.94	0.82	/	/	/	/	/
	Univ. students, USA^b^	Convenience	n10 = 1004(Cameron and Kagee, 2025)	0.87	0.89	0.75	0.84	0.84	/	/	/
	General pop., USA	Stratified: gender & age	n11 = 1004(Chan et al., 2024)	/	/	/	/	0.96	0.066	0.975	0.970
French	General pop., FR, BE	Convenience	n1 = 305(Mouguiama-Daouda et al., 2022)	0.90	0.84	0.82	/	/	0.07	0.92	0.91
	General pop., FR, BE	Conveni ence	n2 = 905(Mouguiama-Daouda et al., 2022)	0.87	0.79	0.81	/	/	0.08	0.89	0.87
German	General pop.	Convenience	n1 = 1134(Wullenkord et al., 2021)	0.89	/	/	/	/	0.084	0.91	0.89
	General pop.	Conveni ence	n2 = 432(Pitron et al., 2024)	0.913	/	/	/	/	/	/	/
Italian	General pop.	Convenience	n1 = 130(Innocenti et al., 2022)	/	/	/	/	/	0.126	0.754	/
	Adolescen ts, IT^b^	Conveni ence	n2 = 189(Di Fabio & Svicher, 2024)	/	0.91	0.79	0.70	0.74	0.064	0.926	0.916
Japanese	General pop.	Stratified: gender & age	n = 1000(Tam et al., 2023)	/	0.93	0.93	/	/	0.134	0.900	/
Korean	General pop.	Convenience	n = 350(Jang et al., 2023)	/	0.85	0.89	/	/	0.120	0.89	0.87
Polish	General pop.	Convenience	n = 603(Larionow et al., 2022)	0.92	0.87	0.89	/	/	0.085	0.936	0.922
Portuguese	General pop.	Convenience	n = 535(Leite et al., 2023)^,b^	/	0.83	0.85	/	/	0.07	0.872	0.856
Slovenian	Age 18 - 24	Convenience	n = 442(Plohl et al., 2023)	/	0.90	0.86	/	/	0.032	0.997	0.996
**Climate Change Distress and Impairment Scale (CC-DIS, *i *= 23), ***S1: Distress (i = 15); S2: Impairment (i = 8)*											
English	General pop.	Convenience	n1 = 447(Hepp et al., 2023)	/	0.914	0.88	/	/	0.072	0.904	0.888
			n2 = 374(Hepp et al., 2023)	/	0.92	0.89	/	/	0.057	0.939	0.930
German	General pop.	Convenience	n1 = 494(Hepp et al., 2023)	/	0.93	0.81	/	/	0.079	0.903	0.888
	General pop.	Online panel	n2 = 2969(König et al., 2024)	/	0.916	0.77	/	/	/	/	/
**Climate Change Distress Scale (CCDS) (*i* = 12), ***S1: climate change anxiety (i = ?); S2: climate change hopelessness (i = ?)*											
English	General pop. & Univ. students, AU	Convenience	n = 257(Searle & Gow, 2010)	0.92	0.92	0.82	/	/	/	/	/
** Climate Change Man Made Disaster Scale (CC-MMDS) (*i *= 16), ***S1: psychological distress (i = 8), S2: change of existing belief systems (i = 8)*											
German	General pop., GE	Convenience	n = 715(Beckord et al., 2024)	0.94	0.93	0.88	/	/	0.096	0.913	0.898
**Climate Change Worry Scale (*i *= 10), ***Unimodal Scale*											
Arabic	General pop., Egypt	Convenience	n = 1300(Hussein et al., 2023)	/	/	/	/	/	/	/	/
English	General pop., USA	Convenience	n1 = 224(Qi et al., 2022)	0.93	/	/	/	/	/	/	/
	Univ. students, USA	Conveni ence	n2 = 600(Stewart, 2021)	0.95	/	/	/	/	0.043	0.99	/
	Univ. students, USA	Convenience	n3 = 54(Stewart, 2021)	0.90	/	/	/	/	/	/	/
	Univ. students, USA	Convenience	n4 = 417(Stewart, 2021)	/	/	/	/	/	/	/	/
	Univ. students, USA	Convenience	n5 = 308(Stewart et al., 2023)	0.95	/	/	/	/	/	/	/
French	General pop., FR	Convenience	n = 442(Shepherd et al., 2024)	0.91	/	/	/	/	0.128	0.895	0.864
Italian	General pop., ITA	Convenience	n = 130(Innocenti et al., 2022)	0.975	/	/	/	/	/	0.91	0.88
Polish	General pop., PL	Convenience	n = 420(Larionow, Gawrych, et al., 2024)	0.93	/	/	/	/	0.043	0.997	0.996
Turkish	Women, TUR	Convenience	n = 321(Demir et al., 2023)	0.97	/	/	/	/	/	/	/
Slovenian	Age 18-24, SVN	Convenience	n = 442(Plohl et al., 2023)	0.96	/	/	/	/	0.035	0.999	0.999
**ECO-ANS-LATAM (*i *= 4),** *Unimodal Scale*											
Spanish	General pop.	Convenience	n = 1907(Mejia et al., 2024)	0.88	/	/	/	/	0.070	0.990	0.990
**Eco-Anxiety Questionnaire (EAQ, *i *= 22), ***S1: Habitual Ecological Worry (i = 13); S2: Negative Consequences of Eco-Anxiety (i =9)*											
French	General pop.	Quotas sampling	n = 1004(Micoulaud-Franchi et al., 2024)	0.934	/	/	/	/	/	/	/
German	General pop.	Convenience	n = 871(Zeier & Wessa, 2024)	/	0.93	0.86	/	/	0.061	0.976	0.974
Hungarian	General pop.	Convenience	n = 1152(Ágoston, Urbán, et al., 2022)	/	0.91	0.86	/	/	0.056	0.972	0.969
**Environmental Distress Scale (EDS, *i *= 81), ***S1: Frequency (i = 10); S2: Observation; (i = 9); S3: Threat (i = 18); S4: Impact (i = 21); S5: Solastalgia (i = 9; *α = 0.9(Tam et al., 2023)), *S6: Actions (i = 14; *α = 0.79*)*											
English	General pop., AUS	Stratified: urban, peri-urban, rural locals	n = 203(Higginbotham et al., 2006)	0.79-0.96	0.92	0.82	0.96	0.94	/	/	/
**EMEA (i = 22), ***S1: Anxiety-depressive manifestations (i = 9), S2: Relational disturbances (i = 7), S3: Ecological obsessions (i = 6)*											
French (Jalin et al., 2025)	General pop., FR	Convenience	n = 429	0.925	0.878	0.82	0.845		0.057	0.93	0.92
**General-Anxiety-Disorder-Climate Version (GAD-7-C, *i* = 7), ***Unimodal Scale*											
German	Patients (Psychosomatic outpatient clinic)	Convenience	n1 = 84(Gebhardt et al., 2023)	0.83	/	/	/	/	/	/	/
	Medical students	Convenience	n2 = 203(Schwaab et al., 2022)	0.83	/	/	/	/	/	/	/
**Hogg Eco-Anxiety Scale (HEAS, *i* = 13)**, *S1: Affective symptoms (i = 4); S2: Rumination (i = 3); S3: Behavioral symptoms (i = 3); S4: Personal impact anxiety (i = 3)*											
Arabic	General pop., LBY	Convenience	n = 829(Ali et al., 2024)	/ /	0.72	0.79	0.68	0.65	0.04	0.99	0.97
English	General pop., AUS	Convenience	n1 = 529(T. L. Hogg et al., 2023)	/	0.88	0.86	0.78	0.86	0.05	0.08	0.98
	Univ. students, AUS	Conveni ence	n2 = 342(T. L. Hogg et al., 2021)	/	0.92	0.90	0.86	0.88	0.08	0.96	0.95
	General pop., AU	Conveni ence	n3 = 530(T. L. Hogg et al., 2024)	/	0.88	0.86	0.78	0.86	/	/	/
	Age 18 - 30, AUS	Convenience	n4 =96(Mathers -Jones & Todd, 2023)	/	/	/	/	/	/	/	/
	General pop., ITA & IND	Convenience	n5= 557(Sharma et al., 2023)	0.940	/	/	/	/	/	/	/
	General pop., AUS	Quota Sampling	n6 = 287(Karl & Stanley, 2024)	/	0.88	0.88	0.82	0.83	0.02	1.0	/
	General pop., UK	Conveni ence	n7 = 501(T. Hogg et al., 2024)	/	0.84	0.79	0.70	0.87	0.05	0.98	0.97
	General pop., UK	Convenience	n8 = 508(T. Hogg et al., 2024)	/	0.90	0.89	0.84	0.90	0.08	0.96	0.95
French	General pop., FRA	Convenience	n = 275(Mathé et al., 2023)	0.91	0.85	0.85	0.73	0.88	0.06	0.97	0.96
German	General pop., GER	Convenience	n1 = 486(Rohn et al., 2025)	/	0.83	0.86	0.71	0.83	0.055	0.983	0.977
	Univ. students, GER	Conveni ence	n2 = 256 (Rohn et al., 2025)	0.91	0.85	0.84	0.81	0.88	0.07	0.96	0.95
Italian	General pop., ITA	Convenience	n1 = 335(94)	/	0.859	0.84	0.781	0.85	0.074	0.956	/
	General pop., ITA	Conveni ence	n2 = 150(Innocen ti et al., 2023)	0.986	/	/	/	/	/	0.683	0.623
Polish	General pop., PL	Convenience	n = 634(Larionow, Mackiewicz, et al., 2024)	/	0.84	0.85	0.79	0.84	0.064	0.961	0.949
Portuguese	General pop.	Convenience	n1 =197(Parreir a & Mouro, 2023)	0.86	/	/	/	/	/	/	/
	Age 18-25, stud.	Conveni ence	n2 = 623(Sampaio et al., 2023)	/	0.847	0.893	0.861	0.9	0.057	0.9	0.9
Spanish	General pop., ARG & ESP	Convenience	n = 1538(Rodríguez Quiroga et al., 2024)	/	0.78	0.81	0.71	0.79	0.04	0.98	0.97
Turkish	Univ. stud. & teachers	Convenience	n1 = 385(Çimşir et al., 2024)	0.87 / /	0.85 /	0.85 /	0.80 /	0.74 /	0.032 / 0.056	0.993	0.983
	Nursing stud.	Convenience	n2 = 609(Er et al., 2024)	/	/	/	/	/	/	/	/
	Univ. stud.	Convenience	n3 = 605(Kabasak al-Cetin, 2023)	/	/	/	/	/	0.056	0.974	/
	General pop.	Convenience	n4 =605(Türkarslan et al., 2023)	/	0.88	0.84	0.82	0.88			/
	General pop.	Convenience	n5 = 289(Türkarslan et al., 2023)	/	/	/	/	/	/	/	0.98
	General pop.	Convenience	n6 = 698(Uzun et al., 2022)	0.91	0.83	0.86	0.84	0.84	0.05 0.06	0.98 0.97	/
**Scale of Solastalgia (SOS, *i* = 10), ***S1: Solace (i = ?); S2: Algia (i = ?)*											
English(Cáceres et al., 2022)	General pop., Chile	Cluster sampling	n = 223	0.901	0.87	0.91	/	/	/	/	/

**Table 3:** COSMIN Criteria for Good Measurement Properties of Patient-Reported Outcome Measures (PROMs), (Mokkink et al., 2024)

Instrument	Reference	Content Validity	Structural Validity	Internal Consistency	Measurement Invariance	Reliability
ASS	(Stanley, 2023)	**( +)**	**( +)**	**( +)**	**( +)**	(?)
BSS	(Christensen et al., 2024)	**( +)**	**( +)**	**( +)**	**( +)**	(?)
CACHI	(Sangervo et al., 2022)	**( +)**	(?)	(-)	(?)	(?)
CCAS	(Clayton & Karazsia, 2020)	**( +)**	( ~)	**( +)**	( ~)	**( +)**
CC-DIS	(Hepp et al., 2023)	**( +)**	(-)	**( +)**	(?)	(?)
CCDS	(Searle & Gow, 2010)	**( +)**	(?)	**( +)**	(?)	(?)
CC-MMDS	(Beckord et al., 2024)	**( +)**	**( +)**	**( +)**	**( +)**	(?)
CCWS	(Stewart, 2021)	**( +)**	( ~)	**( +)**	**( +)**	**( +)**
EAQ	(Ágoston et al., 2022a, b)	**( +)**	**( +)**	**( +)**	(?)	(?)
ECO-ANS-LATAM	(Mejia et al., 2024)	**( +)**	**( +)**	**( +)**	(?)	(?)
EDS	(Higginbotham et al., 2006)	**( +)**	(?)	**( +)**	(?)	(?)
EMEA	(Jalin et al., 2025)	**( +)**	**( +)**	**( +)**	(?)	**( +)**
GAD-7-C	(Schwaab et al., 2022)	**( +)**	(?)	**( +)**	(?)	(?)
HEAS	(T. L. Hogg et al., 2021)	**( +)**	**( +)**	**( +)**	**( +)**	( ~)
SOS	(Cáceres et al., 2022)	**( +)**	**( +)**	**( +)**	(?)	(?)
Note: Measurement Error was not assessed by any of the studies. Criterion validity and responsiveness were excluded by us as no standard instrument against which one could compare exists						

*ASS* = *Anticipatory Solastalgia Scale, BSS* = *Brief Solastalgia Scale, CACHI* = *Climate-Anxiety-Climate-Hope-Index, CCAS* = *Climate Change Anxiety Scale, CC-DIS* = *Climate Change Distress and Impairment Scale, CCDS* = *Climate Change Distress Scale, CC-MMDS* = *Climate Change Version Man Made Disaster-Related Distress Scale, CCWS* = *Climate Change Worry Scale, EAQ* = *Eco Anxiety Questionnaire, ECO-ANS-LATAM* = *no full version is given by the authors, EDS* = *Environmental Distress Scale, EMEA* = *Échelle de mesure de l’éco-anxiété, GAD-7-C* = *Generalized Anxiety Disorder-Climate Version, HEAS* = *Hogg Eco-Anxiety Scale, SOS* = *Scale of Solastalgia*

Overall, many psychometric properties were not assessed by the initial validation studies, nor by following publications. Most notably, measurement error was not assessed in any of the studies. If measurement properties were reported, they usually met the criteria for good psychometric quality. The Anticipatory Solastalgia Scale (Stanley, 2023), the Brief Solastalgia Scale (Christensen et al., 2024), the Climate Change Version of the Man-Made Disaster Scale (Beckord et al., 2024), the Climate Change Worry Scale (Stewart, 2021), the EMEA Scale (Jalin et al., 2025), and the Hogg Eco-Anxiety Scale (T. L. Hogg et al., 2021) showed good measurement properties for most indicators. Of these, the Hogg-Eco-Anxiety Scale stands out because it has been validated several times in different countries and settings. For the Climate Change Anxiety Scale (Clayton & Karazsia, 2020), on the other hand, the factorial structure could oftentimes not be replicated in other samples, and measurement invariance could not be established.

### Correlations of Eco-Distress Questionnaires with Mental Health Questionnaires and potential moderators

In total, *n* = 229 correlation coefficients from *k* = 51 studies were included in our review. All correlation coefficient were obtained cross-sectional. Of these, *n* = 87 reported correlations with anxiety, *n* = 72 with depression, *n* = 51 with stress, and *n* = 19 with well-being. The DASS-21 and its short from the DASS-8 (anxiety, depression, stress), the GAD-7 (anxiety), the PHQ-9 (depression), and the combined short form of GAD-7 and PHQ-9, the PHQ-4 (stress), were used most often to assess mental health impairments. Overall, correlations with eco-distress ranged between *-0.07* < *r* > *0.70* for anxiety, *0.09* < *r* > *0.65* for depression, *− 0.24* < *r* > *0.70* for stress, and *-0.29* < *r* > *0.33* for well-being. Correlations for well-being were reversed before entering them into the analysis. A table detailing all correlations is provided as Online Appendix D.

In a null-model without moderators, there was a moderate positive correlation of eco-distress and mental health impairments, *r* = 0.32, 95% CI [0.27; 0.37], *p* < 0.001. Between-study variance was* τ*^*2*^ = 0.0359. Heterogeneity was significant (*Q* = 3691.17, *p* < 0.001), with 95% of the variance due to between-study differences (*I*^*2*^ = 0.9511). Thus, the influence of moderating effects seemed highly plausible. First, eco-distress questionnaire was entered as a moderator. The resulting model showed a significantly better fit than the null-model in a likelihood ratio test (*p* < 0.001). However, it did not explain a relevant share of the variance in effect sizes (pseudo *R*^*2*^ = -0.08). Thus, the moderator was not retained. As the type of eco-distress questionnaire was at the core of our analysis, we still tested whether there were significant differences in effect size between questionnaires when including category of mental health questionnaire as an additional moderator. There were no significant differences in effect size between eco-distress questionnaires (0.14 < *p* > 0.98).

**Figure 2:**
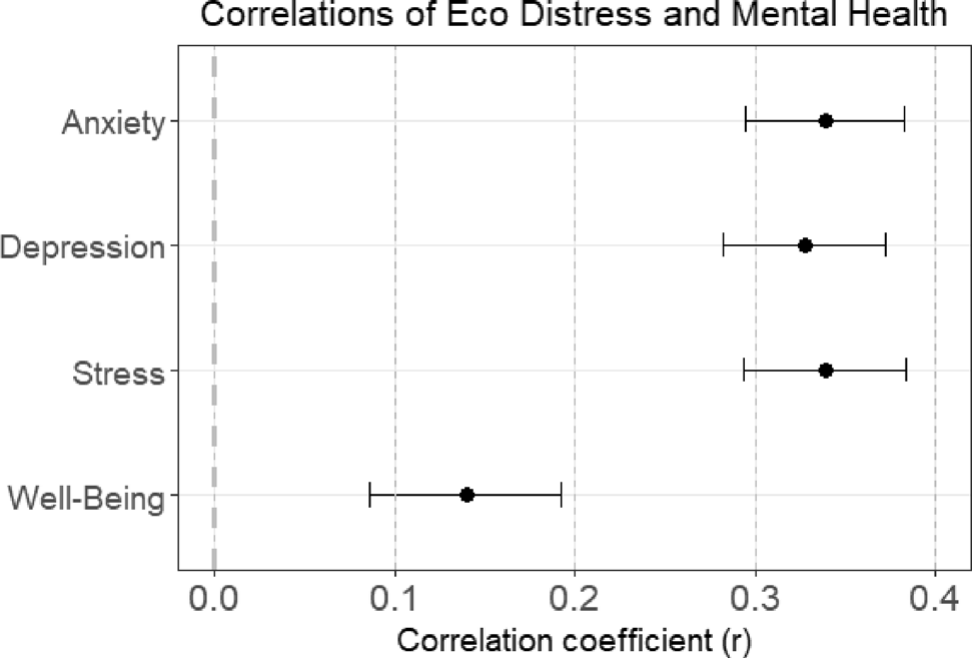
Forest plot of the pooled correlation coefficients with confidence intervals of eco-distress and mental health impairments from the moderated meta-analysis with mental health category (anxiety, depression, stress, and well-being) as a moderator. The data the figure is based on is provided as Online Appendix D.

Next, we tested the category of mental health questionnaire as a moderator. The resulting model showed a significantly better fit than the null-model in a likelihood ratio test (*p* < 0.001), and it explained 20% of between-study heterogeneity (pseudo *R*^*2*^ = 0.20). We tested the factor levels for significant differences. Only well-being differed significantly from the other three categories (all *p* < 0.001), there were no significant differences in effect sizes between anxiety, depression, and stress (all *p* > 0.05). Effect sizes per category of mental health questionnaire are shown in Fig. 2.

**Figure 3:**
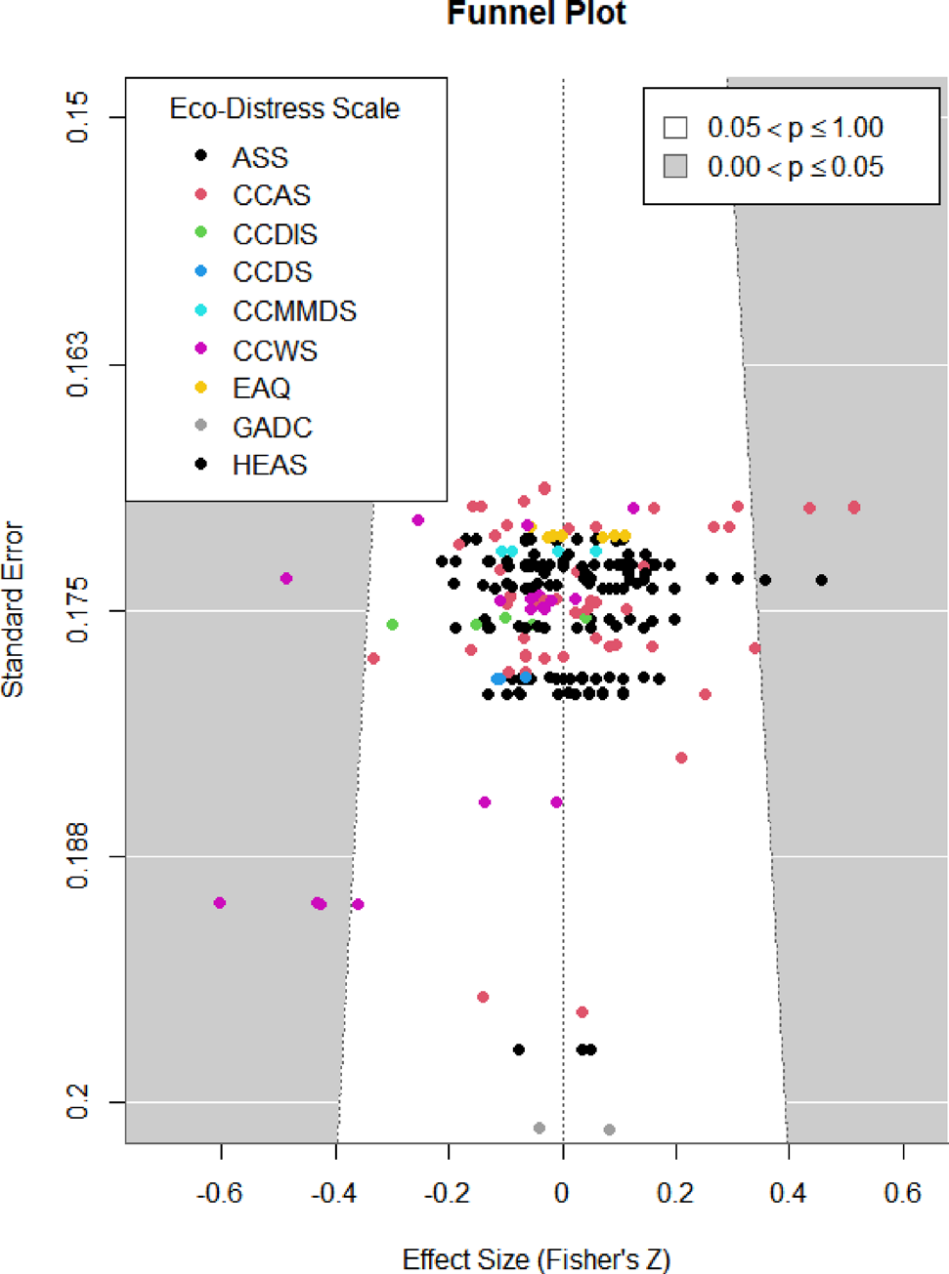
Funnel Plot of the moderated meta-analysis of the correlation of eco-distress and mental health impairments from the moderated meta-analysis with mental health category (anxiety, depression, stress, and well-being) as a moderator. The eco-distress questionnaire the effect size was extracted from is indicated in color. *ASS* Anticipatory Solastalgia Scale; *CCAS* Climate Change Anxiety Scale; *CC-DIS* Climate Change Distress and Impairment Scale; *CCDS* Climate Change Distress Scale; *CC-MMDS* Climate Change Version Man Made Disaster-Related Distress Scale; *CCWS* Climate Change Worry Scale; *EAQ* Eco Anxiety Questionnaire; *GAD-7-C* Generalized Anxiety Disorder-Climate Version; *HEAS* Hogg Eco-Anxiety Scale.

Regarding study characteristics, region improved model fit (*p* < 0.001), but did not explain a relevant share of the variance (pseudo *R*^*2*^ = *− *0.26). Mean age and percentage of female participants both did not improve model fit (*p* = 0.47; *p* = 0.98). Regarding item overlap with a generalized anxiety disorder or a depressive disorder as defined by the ICD-11, the ratings ranged from 0 to 100% between scales. The percentages of item overlap are provided in Appendix D. Percentage of item overlap with anxiety was tested as a moderator for all correlations of eco-distress questionnaires with anxiety questionnaires. The model showed a significantly better fit than the null-model (*p* < 0.001), but it did not explain a relevant share of the variance (pseudo *R*^*2*^ = *− *0.11). Percentage of item overlap with depression was tested as a moderator for all correlations of eco-distress questionnaires with depression questionnaires. The moderator was not significant (*p* = 0.96). Thus, only category of mental health questionnaire was retained as a significant moderator in our analysis. However, between-study heterogeneity was still high for this model (*I*^*2*^ = 0.9241). Figure 3 displays the funnel plot for the respective model.

### Synthesis of Results

**Figure 4:**
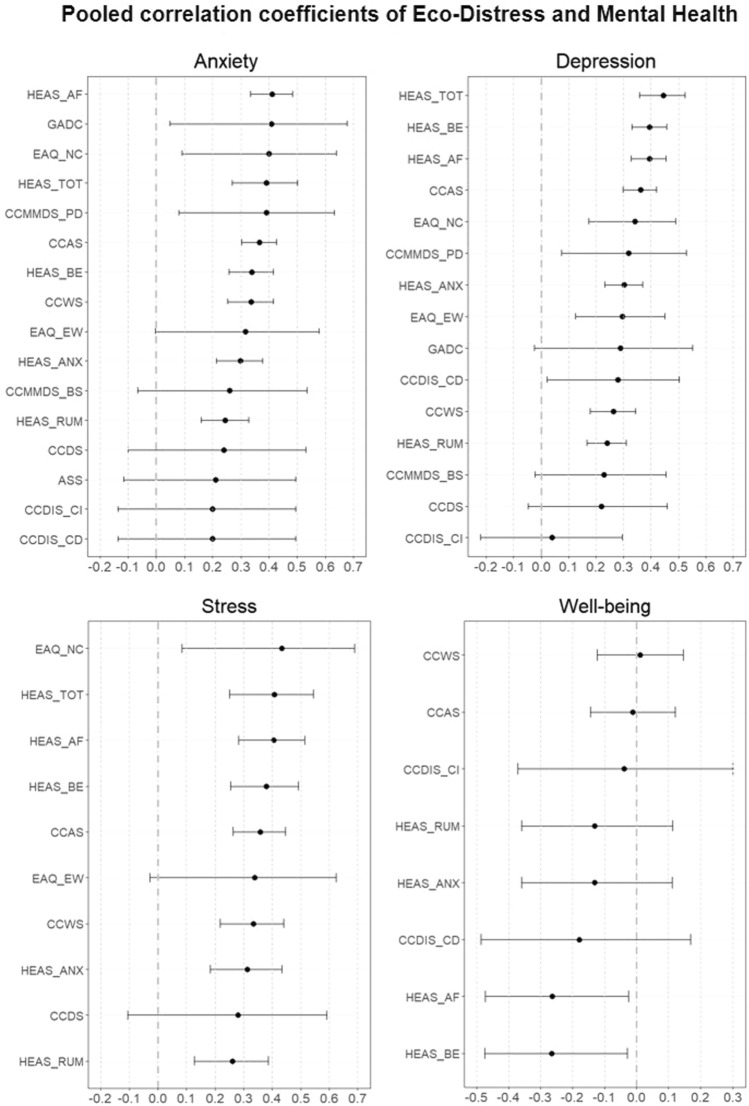
Forest plot of the pooled correlation coefficients with confidence intervals from the moderated meta-analysis with eco-distress questionnaire and mental health category (anxiety, depression, stress, and well-being) as moderators. The data the figure is based on is provided as Appendix D. *ASS* Anticipatory Solastalgia Scale; *CCAS* Climate Change Anxiety Scale; *CC-DIS_CD* Climate Change Distress and Impairment Scale, Subscale Distress; *CC-DIS_CI* Climate Change Distress and Impairment Scale, Subscale Impairment; *CCDS* Climate Change Distress Scale; *CC-MMDS_BS* Climate Change Version Man Made Disaster-Related Distress Scale, Subscale Belief System; *CC-MMDS_PD* Climate Change Version Man Made Disaster-Related Distress Scale, Subscale Psychological Distress; *CCWS* Climate Change Worry Scale; *EAQ_EW* Eco Anxiety Questionnaire, Subscale Ecological Worry; *EAQ_NC* Eco Anxiety Questionnaire, Subscale Negative Cognitions; *GAD-7-C* Generalized Anxiety Disorder-Climate Version; *HEAS_AF* Hogg Eco-Anxiety Scale, Subscale Affective Symptoms; *HEAS_ANX* Hogg Eco-Anxiety Scale, Subscale Anxiety; *HEAS_BE* Hogg Eco-Anxiety Scale, Subscale Behavioral Symptoms; *HEAS_RUM* Hogg Eco-Anxiety Scale, Subscale Rumination.

The first methodological reason for heterogeneity in correlations of eco-distress and mental health impairments presumed by us were large measurement errors due to poor psychometric quality. To test this claim, we visualized the confidence intervals per eco-distress questionnaire in Fig. 4, even though type of questionnaire did not show to be a significant moderator. As can be seen, no clear pattern of larger confidence intervals (Fig. 4) for eco-distress questionnaires of poorer psychometric quality (Table 3) emerges. For example, the Climate Change Worry Scale shows good psychometric properties, but the confidence intervals around the pooled correlation coefficients are larger than for most other eco-distress questionnaires included in the review. Therefore, no systematic relationship of heterogeneity in correlations with psychometric quality is apparent in our data.

The second methodological reason for heterogeneity in correlations of eco-distress and mental health impairments presumed by us were different underlying concepts of eco-distress. If this was the case, correlations of eco-distress with mental health impairments would vary systematically depending on the instrument employed to measure eco-distress. However, the type of eco-distress questionnaire did not explain a relevant share of the variance in effect sizes. The third methodological reason for heterogeneity in correlations of eco-distress and mental health impairments presumed by us were sample and study characteristics. However, none of the potential moderators tested by us (region, age, gender) explained a relevant share of the variance. The fourth methodological reason for heterogeneity in correlations of eco-distress and mental health impairments presumed by us was percentage of item overlap with an anxiety disorder and a depressive disorder. However, neither moderator explained a relevant share of the variance.

## Discussion

Our review was the first to combine aspects of methodology and correlations with mental health impairments. In contrast to previous approaches, we focused on significant differences, not on finding commonalities between the constructs to synthesize an overarching definition of eco distress. In combining the analysis of the relationship of eco-distress and mental health impairments with an analysis of methodology, study and sample characteristics, we were able to explore systematic relationships of these constructs. Previous reviews showed that there is considerable variance in the concepts underlying different eco-distress questionnaires and in the size of the correlation of eco-distress and mental health impairments. Through our approach, we could show that neither the variance in the concepts, nor in psychometric quality systematically influenced the correlation of eco-distress and mental health impairments. However, we could also not discern any other variables influencing the size of the correlation, except for the finding that correlations of eco-distress and well-being are significantly lower than those with anxiety, depression, or stress. Even after including this moderator, 92% of the between-study heterogeneity remained unexplained.

Regarding the underlying concept of eco-distress, the questionnaires included in our review all assessed some component of anxiety or worry, but the conceptualization differed. For example, while the Climate Change Anxiety Scale (Clayton & Karazsia, 2020) assessed a clinically significant response, the Climate Change Distress and Impairment Scale (Hepp et al., 2023) assessed a non-pathological response. In addition, some instruments focused solely on anxiety, while others included other emotions such as depression, and yet other instruments did not differentiate and assessed psychological distress in general. Finally, while some instruments focused solely on the emotional response to the threat of climate change, others included cognitive, behavioral, and physiological indicators, as well. In sum, the conceptualization of eco-distress in the instruments included in our review mirrors the variety in the definition of the concept (Coffey et al., 2021). Moreover, the psychometric quality of the instruments was mixed. Overall, if measurement properties were reported, they tended to be acceptable. For many of the instruments, however, no information could be retrieved from the studies included in our review, especially regarding measurement invariance, (re-test) reliability, and measurement error.

However, these substantial qualitative differences in the underlying concepts of eco-distress, as well as the varying psychometric quality of the validation studies published so far, did not translate into systematic variations of the correlation of eco-distress and mental health. Moreover, none of the study-related moderators tested by us explained a relevant share of the high between-study heterogeneity. Two explanations for this finding are possible: either the high between-study heterogeneity is explained by other moderatos not tested by us; or there is a random variability in study results. The substantial range of the correlation coefficients (*− *0.07 < *r* > 0.70 for anxiety, 0.09 < *r* > 0.65 for depression, *− *0.24 < *r* > 0.70 for stress, and *− *0.29 < *r* > 0.33 for well-being) suggests the latter. These findings are mirrored by the results of a meta-analysis on the correlation of the Climate Change Anxiety Scale and well-being: the authors reported a reversed effect size similar to our findings, with *r* = *− *0.296, 95% CI [*− *0.36; -0.23] (our results: *r* = 0.32, 95% CI [0.27; 0.37]) and high levels of between-study heterogeneity (Gago et al., 2024). These results call for a thorough methodological re-evaluation of the existing instruments and methodologically sound validation studies with representative samples sufficient in size.

Of note, the percentage of item overlap of eco-distress questionnaires with syndromes of anxiety and depression was also not a significant moderator. Thus, variations in correlations of eco-distress with mental health impairments are not a result of the shared number of items assessing the same emotion or behavior. This findings supports the definition of eco-distress as an independent construct. If percentage of item overlap had moderated the size of the correlation, this would have meant that the correlation of eco-distress and mental health impairments was at least partially an artefact of similarities in measurement. In contrast, our data show that although there are commonalities between eco-distress and mental health impairments, it is not possible to simply conclude an individual’s level of eco-distress from their level of depression or anxiety.

Moreover, the size of the correlation of eco-distress with questionnaires assessing anxiety, depression, or stress did not differ significantly. Such a consistency in the size of the correlation with questionnaires assessing different aspects of mental health impairments suggests a common underlying factor causing small-to-medium positive correlations between the constructs. Various theories of mental health offer an explanation for the correlation between different aspects of mental health impairments. Following the conceptualization laid out by the Research Domain Criteria (RDoC) framework, these correlations might be due to a shared neurobiological basis causing a certain amount of non-syndrome-specific psychosocial distress (Williams et al., 2024). Following the conceptualization of the network approach, these correlations might be due to the inclusion of interacting and reciprocally reinforcing symptoms (Borsboom & Cramer, 2013). The conclusion from both conceptualizations would be that treatment approaches which have been shown to be effective in reducing symptoms of anxiety, depression, or general stress will likely be effective in reducing symptoms of eco-distress as well, at least for the set of symptoms of eco-distress shared with other concepts of mental health impairments.

### Limitations

Due to our initial decision to include only quantitative studies, we excluded possible valuable sources of qualitative research which might clarify the definition of eco-distress. However, as our aim was to provide an applicable overview of which instruments are available, the inclusion of such studies was deemed to be outside of our focus. Furthermore, we selected only instruments which included a variation of anxiety in their definition of eco-distress. Thereby, we excluded instruments specifically assessing other aspects of the emotional response to climate change, such as eco-guilt or ecological sadness (Ágoston et al., 2022a, b). Hereby, we aimed to offer a review precise enough in its focus to still be of use. Regarding our choice to operationalize item overlap between eco-distress questionnaires and syndromes of anxiety and depression, we acknowledge that there is no standardized method to compare questionnaires for similarity. We adopted a rather broad definition of item overlap, as we scored items to be overlapping if they named the same emotion or behavior, regardless of its context or severity. Results might differ if based on alternative approaches. Finally, we did not assess the psychometric quality of the mental health questionnaires used to assess anxiety, depression, stress and well-being in the studies included in our review. While most of the instruments are well-established in mental health research for anxiety and depression, results regarding stress and well-being should therefore be interpreted with caution, as measurement error could be caused by the psychometric quality of the questionnaire assessing stress or well-being, rather than by the eco-distress questionnaire.

### Conclusions and Future Directions

In conclusion, there is a large variation in correlations of eco-distress and mental health impairments which could not be fully explained by any of the moderators tested by us and which seemed to be independent of the psychometric quality of the eco-distress questionnaire. Therefore, results from single studies on the correlation of eco-distress and mental health impairments should only be interpreted with great caution. Moreover, due to the high amount of missing information on psychometric quality, it is not possible to recommend single questionnaires for clinical practice or future research. While the overall correlation between eco-distress and mental health impairments of *r* = 0.32 fits well with previous research, the results of eco-distress questionnaires on an individual level cannot be used as a screening tool or cut-off criterion, seeing the high range in correlations reported in the literature. Thus, interested clinicians are well advised to understand eco-distress questionnaires descriptively, in the sense that they might use them to screen whether clients report any symptoms of eco-distress, and then explore these symptoms further in a clinical interview. If used this way, clinicians could choose the eco-distress questionnaire whose underlying concept is relevant to them. Such a decision could be guided by our overview of the eco-distress questionnaires. Moreover, future research might focus on the psychometric evaluation and validation of existing instruments, rather than on the development of new ones. As there are no significant differences between questionnaires which relied on different concepts of eco-distress, but large ranges in the size of the correlations per instrument, it seems sensible to explore these variations in more depth.

## Supplementary Information

Below is the link to the electronic supplementary material.

Supplementary file 1 (DOCX 16 kb)

Supplementary file 2 (DOCX 26 kb)

Supplementary file 3 (DOCX 26 kb)

Supplementary file 4 (DOCX 155 kb)

## Data Availability

All data generated or analyzed during this study are included in this published article and its supplementary information files.
