# Supplementary material for: Systematic Review of the Links between Eco-Distress and Mental Health
Source: Ecohealth. 2025 Dec 2;23(2):270–89. doi: 10.1007/s10393-025-01769-z (PMC13287264; doi:10.1007/s10393-025-01769-z)
Supplement: Supplementary file 1 — (DOCX 16 kb) [file 10393_2025_1769_MOESM1_ESM.docx]

**Supplement S1: Search Terms**

“eco-anxiety” OR “eco anxiety” OR “climate anxiety” OR “climate change anxiety” OR “climate distress” OR “eco-distress” OR “eco distress” OR “eco-emotions” OR “eco emotions” OR “climate emotions” OR “psychoterratic syndrome” OR “climate change awareness” OR “psychological impact of climate change” OR solastalgia OR “climate worry” OR “climate change-related worry” OR “ecosystem distress syndrome” OR “eco anger” OR “eco depression” OR “eco guilt”

AND

scale OR inventory OR measure* OR questionnaire* OR assessment* OR correlates OR instrument* OR survey

SEARCH: 15.03.2025
