# Supplementary material for: Systematic Review of the Links between Eco-Distress and Mental Health
Source: Ecohealth. 2025 Dec 2;23(2):270–89. doi: 10.1007/s10393-025-01769-z (PMC13287264; doi:10.1007/s10393-025-01769-z)
Supplement: Supplementary file 2 — (DOCX 26 kb) [file 10393_2025_1769_MOESM2_ESM.docx]

Supplement S1. COSMIN Risk of Bias Checklist for systematic reviews of Patient-Reported Outcome Measures (PROMs), (Mokkink et al., 2024).

| Instrument | Reference | PROM development | Content Validity | Structural Validity | Internal Consistency | Measurement Invariance | Reliability | Measurement Error | Construct Validity |
| --- | --- | --- | --- | --- | --- | --- | --- | --- | --- |
| ASS | (Stanley, 2023) | Inadequate | Inadequate | **Very Good** | **Very Good** | **Very Good** | Inadequate | Inadequate | **Very Good** |
| **BSS** | (Christensen et al., 2024) | Inadequate | Inadequate | **Very Good** | **Very Good** | **Very Good** | Inadequate | Inadequate | **Very Good** |
| CACHI | (Sangervo et al., 2022) | Inadequate | Inadequate | Inadequate | Inadequate | Inadequate | Inadequate | Inadequate | **Adequate** |
| CCAS | (Clayton & Karazsia, 2020) | Inadequate | Inadequate | **Very Good** | **Very Good** | Inadequate | Inadequate | Inadequate | **Very Good** |
| CC-DIS | (Hepp et al., 2023) | Inadequate | Inadequate | **Very Good** | **Very Good** | Inadequate | Inadequate | Inadequate | **Very Good** |
| CCDS | (Searle & Gow, 2010) | Inadequate | Inadequate | **Very Good** | **Very Good** | Inadequate | Inadequate | Inadequate | **Adequate** |
| **CC-MMDS** | (Beckord et al., 2024) | Inadequate | Inadequate | **Very Good** | **Very Good** | **Very Good** | Inadequate | Inadequate | **Very Good** |
| **CCWS** | (Stewart, 2021) | Inadequate | Inadequate | **Very Good** | **Very Good** | **Very Good** | **Very Good** | Inadequate | **Very Good** |
| **EAQ** | (Ágoston et al., 2022) | **Very Good** | Inadequate | **Very Good** | **Very Good** | Inadequate | Inadequate | Inadequate | **Adequate** |
| ECO-ANS-LATAM | (Mejia et al., 2024) | Inadequate | Inadequate | **Very Good** | **Very Good** | Inadequate | Inadequate | Inadequate | Inadequate |
| EDS | (Higginbotham et al., 2006) | Inadequate | Inadequate | Inadequate | **Very Good** | Inadequate | **Very Good** | Inadequate | **Adequate** |
| **EMEA** | (Jalin et al., 2025) | **Very Good** | **Very Good** | **Very Good** | **Very Good** | Inadequate | **Very Good** | Inadequate | **Very Good** |
| GAD-7-C | (Schwaab et al., 2022) | Inadequate | Inadequate | Inadequate | **Very Good** | Inadequate | Inadequate | Inadequate | **Very Good** |
| **HEAS** | (Hogg et al., 2021) | Doubtful | Inadequate | **Very Good** | **Very Good** | Inadequate | **Very Good** | **Adequate** | **Very Good** |
| SOS | (Cáceres et al., 2022) | Inadequate | Inadequate | **Very Good** | **Very Good** | Inadequate | Inadequate | Inadequate | **Very Good** |

Note. Criterion validity and Responsiveness were not assessed, as no gold standard to assess eco-distress exists so far.

Abbreviations: ASS = Anticipatory Solastalgia Scale; BSS = Brief Solastalgia Scale; CACHI = Climate-Anxiety-Climate-Hope-Index; CCAS = Climate Change Anxiety Scale; CC-DIS = Climate Change Distress and Impairment Scale; CCDS = Climate Change Distress Scale; CC-MMDS = Climate Change Version Man Made Disaster-Related Distress Scale; CCWS = Climate Change Worry Scale; EAQ = Eco Anxiety Questionnaire; ECO-ANS-LATAM = no full version is given by the authors; EDS = Environmental Distress Scale; EMEA = Échelle de mesure de l’éco-anxiété; GAD-7-C = Generalized Anxiety Disorder-Climate Version; HEAS = Hogg Eco-Anxiety Scale; SOS = Scale of Solastalgia
