# Supplementary material for: Systematic Review of the Links between Eco-Distress and Mental Health
Source: Ecohealth. 2025 Dec 2;23(2):270–89. doi: 10.1007/s10393-025-01769-z (PMC13287264; doi:10.1007/s10393-025-01769-z)
Supplement: Supplementary file 4 — (DOCX 155 kb) [file 10393_2025_1769_MOESM4_ESM.docx]

Appendix D:

| **Source** | **Scale_Eco** | **Scale_Psych** | **Psych_Cat** | **n** | **MW age** | **SD age** | **%female** | **% dep** | **% anx** | **cor** |
| --- | --- | --- | --- | --- | --- | --- | --- | --- | --- | --- |
| (Stanley, 2023) | ass | BPSWQ | anx | 509 | 41,82 | 12,5 | 49,9 | 0,2 | 0,2 | 0,16 |
|  | ass | BPSWQ | anx | 493 | 36,34 | 13,00 | 50,5 | 0,2 | 0,2 | 0,26 |
| (Latkin et al., 2022) | bccds | CES-D10 | dep | 775 | 40,84 | 11,91 | 53,42 | 0,2 | 0,4 | 0,239 |
| (Asgarizadeh et al., 2023) | ccas | GAD-7 | anx | 323 | 54,60 | 16,7 | 64,7 | 0,46 | 0,23 | 0,6 |
| (Cameron & Kagee, 2025) | ccas | PHQ-9 | dep | 343 | - | - | - | 0,46 | 0,23 | 0,378 |
|  | ccas | GAD-7 | anx | 343 | - | - | - | 0,46 | 0,23 | 0,279 |
| (Chan et al., 2024) | ccas | GAD-7 | anx | 1004 | - | - | 51,7 | 0,46 | 0,23 | 0,35 |
|  | ccas | PHQ-8 | dep | 1004 | - | - | 51,7 | 0,46 | 0,23 | 0,38 |
|  | ccas | GAD-7 | anx | 1009 | - | - | 48,8 | 0,46 | 0,23 | 0,55 |
|  | ccas | PHQ-8 | dep | 1009 | - | - | 48,8 | 0,46 | 0,23 | 0,56 |
| (Clayton  & Karazsia, 2020) | ccas | PHQ-4 | stress | 197 | - | - | 40,6 | 0,46 | 0,23 | 0,51 |
| (Coates et al., 2024) | ccas | DASS-21 | anx | 256 | 28,2 | 14,6 | 62,2 | 0,46 | 0,23 | 0,54 |
| (Feather & Williams, 2022a) | ccas | GAD-7 | anx | 401 | - | - | 41,9 | 0,46 | 0,23 | 0,37 |
|  | ccas | K-10 | stress | 401 | - | - | 41,9 | 0,46 | 0,23 | 0,36 |
| (Feather & Williams, 2022b) | ccas | PHQ-4 | stress | 771 | 33,0 | 11,85 | 48,0 | 0,46 | 0,23 | 0,17 |
| (Fekih-Romdhane et al., 2024) | ccas | DASS-8 | stress | 596 | 23,46 | 4,12 | 63,9 | 0,46 | 0,23 | 0,46 |
|  | ccas | DASS-8 | stress | 596 | 23,46 | 4,12 | 63,9 | 0,46 | 0,23 | 0,24 |
|  | ccas | DASS-8 | anx | 596 | 23,46 | 4,12 | 63,9 | 0,46 | 0,23 | 0,37 |
|  | ccas | DASS-8 | dep | 596 | 23,46 | 4,12 | 63,9 | 0,46 | 0,23 | 0,35 |
| (Hajek & König, 2023) | ccas | GAD-7 | anx | 3091 | 46,5 | 15,3 | 49,5 | 0,46 | 0,23 | 0,31 |
|  | ccas | PHQ-9 | dep | 3091 | 46,5 | 15,3 | 49,5 | 0,46 | 0,23 | 0,3 |
| (Hussein et al., 2023) | ccas | WHOQOL-BREF | wb | 1300 | 26,93 | 10,16 | 54,7 | 0,46 | 0,23 | -0,293 |
| (Larionow et al., 2022) | ccas | PHQ-4 | stress | 106 | - | - | 47,6 | 0,46 | 0,23 | 0,21 |
| (Lau et al., 2025) | ccas | PHQ-2 | anx | 1567 | 27,83 | 11,63 | 72,0 | 0,46 | 0,23 | 0,194 |
|  | ccas | PHQ-2 | dep | 1567 | 27,83 | 11,63 | 72,0 | 0,46 | 0,23 | 0,196 |
|  | ccas | WHO-5 | wb | 1567 | 27,83 | 11,63 | 72,0 | 0,46 | 0,23 | -0,073 |
| (Lukacs et al., 2023) | ccas | K-6 | stress | 1553 | - | - | 46,4 | 0,46 | 0,23 | 0,7 |
| (Lutz et al., 2023) | ccas | DASS-21 | anx | 308 | 20,15 | 3,95 | 78,6 | 0,46 | 0,23 | 0,34 |
|  | ccas | DASS-21 | dep | 308 | 20,15 | 3,95 | 78,6 | 0,46 | 0,23 | 0,37 |
|  | ccas | DASS-21 | stress | 308 | 20,15 | 3,95 | 78,6 | 0,46 | 0,23 | 0,38 |
|  | ccas | SLS | wb | 308 | 20,15 | 3,95 | 78,6 | 0,46 | 0,23 | 0,02 |
| (Lykins et al., 2024) | ccas | CWS | anx | 103 | 30,84 | 4,87 | 100,,0 | 0,46 | 0,23 | 0,31 |
| (Mouguiama-Daouda et al., 2022) | ccas | GAD-7 | anx | 305 | 30,8 | 11,32 | 72,13 | 0,46 | 0,23 | 0,02 |
|  | ccas | BDI-II | dep | 305 | 30,8 | 11,32 | 72,13 | 0,46 | 0,23 | 0,3 |
| (Parmentier et al., 2024) | ccas | STAI-Y | anx | 431 | 37,6 | 14,6 | 71,7 | 0,46 | 0,23 | 0,25 |
| (Plohl et al., 2023) | ccas | DASS-21 | anx | 442 | 21,57 | 1,67 | 75,8 | 0,46 | 0,23 | 0,3 |
|  | ccas | DASS-21 | stress | 442 | 21,57 | 1,67 | 75,8 | 0,46 | 0,23 | 0,31 |
|  | ccas | MHC-SD | wb | 442 | 21,57 | 1,67 | 75,8 | 0,46 | 0,23 | -0,05 |
| (Reyes et al., 2023) | ccas | MHI-38 | stress | 433 | 20,4 | 1,60 | 66,5 | 0,46 | 0,23 | 0,39 |
|  | ccas | MHI-38 | wb | 433 | 20,4 | 1,60 | 66,5 | 0,46 | 0,23 | -0,05 |
| (Schwartz et al., 2022) | ccas | PHQ-8 | dep | 284 | 23,17 | 3,87 | 78,9 | 0,46 | 0,23 | 0,24 |
|  | ccas | GAD-7 | anx | 284 | 23,17 | 3,87 | 78,9 | 0,46 | 0,23 | 0,28 |
| (Shareeff et al., 2024) | ccas | DASS-21 | dep | 1500 | - | - | 50,0 | 0,46 | 0,23 | 0,65 |
|  | ccas | DASS-21 | anx | 1500 | - | - | 50,0 | 0,46 | 0,23 | 0,7 |
|  | ccas | DASS-21 | stress | 1500 | - | - | 50,0 | 0,46 | 0,23 | 0,58 |
| (Shepherd et al., 2024) | ccas | DASS-21 | dep | 442 | 32,45 | 12,50 | 82,1 | 0,46 | 0,23 | 0,37 |
|  | ccas | DASS-21 | anx | 442 | 32,45 | 12,50 | 82,1 | 0,46 | 0,23 | 0,31 |
|  | ccas | DASS-21 | stress | 442 | 32,45 | 12,50 | 82,1 | 0,46 | 0,23 | 0,33 |
| (Thomson & Roach, 2023) | ccas | DASS-21 | dep | 327 | 24,30 | 9,19 | 72,17 | 0,46 | 0,23 | 0,46 |
|  | ccas | DASS-21 | anx | 327 | 24,30 | 9,19 | 72,17 | 0,46 | 0,23 | 0,41 |
|  | ccas | DASS-21 | stress | 327 | 24,30 | 9,19 | 72,17 | 0,46 | 0,23 | 0,42 |
| (Wagner & Witthöft, 2024) | ccas | PHQ-2 | dep | 415 | 32,59 | 12,35 | 66,9 | 0,46 | 0,23 | 0,365 |
|  | ccas | PHQ-2 | anx | 415 | 32,59 | 12,35 | 66,9 | 0,46 | 0,23 | 0,436 |
| (Whitmarsh et al., 2022) | ccas | GAD-7 | anx | 891 | 47,1 | - | 53,0 | 0,46 | 0,23 | 0,23 |
| (Wullenkord et al., 2021) | ccas | PHQ-4 | stress | 1011 | 43,91 | 13,97 | 51,14 | 0,46 | 0,23 | 0,25 |
| (Hepp et al., 2023) | ccdis_cd | GAD-7 | anx | 374 | 30,71 | 9,18 | 50,3 | 0,33 | 0,27 | 0,2 |
|  | ccdis_cd | BDI-II | dep | 374 | 30,71 | 9,18 | 50,3 | 0,33 | 0,27 | 0,28 |
|  | ccdis_cd | EUROHIS-QOL | wb | 374 | 30,71 | 9,18 | 50,3 | 0,33 | 0,27 | -0,18 |
|  | ccdis_ci | GAD-7 | anx | 374 | 30,71 | 9,18 | 50,3 | 0,38 | 0,25 | 0,2 |
|  | ccdis_ci | BDI-II | dep | 374 | 30,71 | 9,18 | 50,3 | 0,38 | 0,25 | 0,04 |
|  | ccdis_ci | EUROHIS-QOL | wb | 374 | 30,71 | 9,18 | 50,3 | 0,38 | 0,25 | -0,04 |
| (Searle & Gow, 2010) | ccds | DASS-21 | anx | 275 | - | - | 61,0 | 0,17 | 0,25 | 0,24 |
|  | ccds | DASS-21 | dep | 275 | - | - | 61,0 | 0,17 | 0,25 | 0,22 |
|  | ccds | DASS-21 | stress | 275 | - | - | 61,0 | 0,17 | 0,25 | 0,28 |
| (Beckord et al., 2024) | ccmmds_bs | PHQ-8 | dep | 715 | 39,14 | 15,59 | 66,15 | 0 | 0 | 0,23 |
|  | ccmmds_bs | GAD-7 | anx | 715 | 39,14 | 15,59 | 66,15 | 0 | 0 | 0,26 |
|  | ccmmds_pd | PHQ-8 | dep | 715 | 39,14 | 15,59 | 66,15 | 0,45 | 0,36 | 0,32 |
|  | ccmmds_pd | GAD-7 | anx | 715 | 39,14 | 15,59 | 66,15 | 0,45 | 0,36 | 0,39 |
| (Hussein et al., 2023) | ccws | WHOQOL-BREF | wb | 1300 | 26,93 | 10,16 | 54,7 | 0,46 | 0,9 | -0,259 |
| (Innocenti et al., 2022) | ccws | DASS-21 | anx | 130 | 35,02 | 10,68 | 53,1 | 0 | 0,9 | -0,071 |
|  | ccws | DASS-21 | dep | 130 | 35,02 | 10,68 | 53,1 | 0 | 0,9 | -0,091 |
|  | ccws | DASS-21 | stress | 130 | 35,02 | 10,68 | 53,1 | 0 | 0,9 | -0,244 |
|  | ccws | PSWQ | anx | 130 | 35,02 | 10,68 | 53,1 | 0 | 0,9 | -0,005 |
| (Larionow, Gawrych, et al., 2024) | ccws | PHQ-2 | anx | 171 | - | - | - | 0 | 0,9 | 0,33 |
|  | ccws | PHQ-2 | dep | 171 | - | - | - | 0 | 0,9 | 0,2 |
| (Plohl et al., 2023) | ccws | DASS-21 | anx | 442 | 21,57 | 1,67 | 75,8 | 0 | 0,9 | 0,32 |
|  | ccws | DASS-21 | stress | 442 | 21,57 | 1,67 | 75,8 | 0 | 0,9 | 0,36 |
|  | ccws | MHC-SD | wb | 442 | 21,57 | 1,67 | 75,8 | 0 | 0,9 | -0,1 |
| (Shepherd et al., 2024) | ccws | DASS-21 | dep | 442 | 32,45 | 12,50 | 82,1 | 0 | 0,9 | 0,31 |
|  | ccws | DASS-21 | anx | 442 | 32,45 | 12,50 | 82,1 | 0 | 0,9 | 0,24 |
|  | ccws | DASS-21 | stress | 442 | 32,45 | 12,50 | 82,1 | 0 | 0,9 | 0,29 |
| (Söder et al., 2025) | ccws | WHO-5 | wb | 1035 | 25,51 | 5,43 | 50,1 | 0 | 0,9 | 0,112 |
|  | ccws | PHQ-8 | dep | 1031 | 25,51 | 5,43 | 50,1 | 0 | 0,9 | 0,272 |
| (Stewart, 2021) | ccws | DASS-21 | anx | 417 | 20,8 | 1,9 | 85,0 | 0 | 0,9 | 0,29 |
|  | ccws | DASS-21 | dep | 417 | 20,8 | 1,9 | 85,0 | 0 | 0,9 | 0,3 |
|  | ccws | DASS-21 | stress | 417 | 20,8 | 1,9 | 85,0 | 0 | 0,9 | 0,31 |
| (Sümen et al., 2025) | ccws | WEMWBS | wb | 503 | 37,74 | 11,84 | 41,2 | 0 | 0,9 | 0,332 |
| (Micoulaud-Franchi et al., 2024) | eaq_ew | HAD | dep | 1004 | 43,47 | 13,41 | 54,1 | 0 | 0,38 | 0,276 |
| (Zeier & Wessa, 2024) | eaq_ew | PHQ-2 | dep | 871 | 49,3 | 15,3 | 56,4 | 0 | 0,38 | 0,313 |
|  | eaq_ew | PHQ-2 | anx | 871 | 49,3 | 15,3 | 56,4 | 0 | 0,38 | 0,317 |
|  | eaq_ew | PHQ-4 | stress | 871 | 49,3 | 15,3 | 56,4 | 0 | 0,38 | 0,338 |
| (Micoulaud-Franchi et al., 2024) | eaq_nc | HAD | dep | 1004 | 43,47 | 13,41 | 54,1 | 0,56 | 0,56 | 0,276 |
| (Zeier & Wessa, 2024) | eaq_nc | PHQ-2 | dep | 871 | 49,3 | 15,3 | 56,4 | 0,56 | 0,56 | 0,407 |
|  | eaq_nc | PHQ-2 | anx | 871 | 49,3 | 15,3 | 56,4 | 0,56 | 0,56 | 0,4 |
|  | eaq_nc | PHQ-4 | stress | 871 | 49,3 | 15,3 | 56,4 | 0,56 | 0,56 | 0,433 |
| (Gebhardt et al., 2023) | gadc | GAD-7 | anx | 84 | 37,64 | 15,11 | 68,0 | 0,14 | 1 | 0,41 |
|  | gadc | PHQ-9 | dep | 84 | 37,64 | 15,11 | 68,0 | 0,14 | 1 | 0,29 |
| (Ali et al., 2024) | heas_af | DASS-8 | dep | 829 | 22,24 | 3,76 | 84,6 | 0 | 1 | 0,38 |
|  | heas_af | DASS-8 | anx | 829 | 22,24 | 3,76 | 84,6 | 0 | 1 | 0,42 |
|  | heas_af | DASS-8 | stress | 829 | 22,24 | 3,76 | 84,6 | 0 | 1 | 0,43 |
| (Çimsir et al., 2024) | heas_af | BSI | dep | 385 | 31,14 | 11,58 | 61,3 | 0 | 1 | 0,4 |
| (Er et al., 2024) | heas_af | DASS-21 | anx | 609 | - | - | 84,2 | 0 | 1 | 0,469 |
|  | heas_af | DASS-21 | dep | 609 | - | - | 84,2 | 0 | 1 | 0,417 |
|  | heas_af | DASS-21 | stress | 609 | - | - | 84,2 | 0 | 1 | 0,475 |
| (Heinzel et al., 2023) | heas_af | DASS-21 | anx | 486 | 29,43 | 10,63 | 73,5 | 0 | 1 | 0,41 |
|  | heas_af | DASS-21 | dep | 486 | 29,43 | 10,63 | 73,5 | 0 | 1 | 0,46 |
|  | heas_af | DASS-21 | stress | 486 | 29,43 | 10,63 | 73,5 | 0 | 1 | 0,43 |
| (T. L. Hogg et al., 2021) | heas_af | DASS-21 | anx | 365 | 19,90 | 3,59 | 79,7 | 0 | 1 | 0,46 |
|  | heas_af | DASS-21 | dep | 365 | 19,90 | 3,59 | 79,7 | 0 | 1 | 0,37 |
|  | heas_af | DASS-21 | stress | 365 | 19,90 | 3,59 | 79,7 | 0 | 1 | 0,42 |
| (T. L. Hogg et al., 2024) | heas_af | GAD-7 | anx | 530 | 39,49 | 16,46 | 63,2 | 0 | 1 | 0,67 |
|  | heas_af | DASS-21 | dep | 530 | 39,49 | 16,46 | 63,2 | 0 | 1 | 0,57 |
|  | heas_af | SLS | wb | 530 | 39,49 | 16,46 | 63,2 | 0 | 1 | -0,28 |
| (T. Hogg et al., 2024) | heas_af | BPSWQ | anx | 501 | 41,89 | 12,08 | 50,1 | 0 | 1 | 0,32 |
|  | heas_af | BPSWQ | anx | 508 | 36,25 | 12,90 | 49,8 | 0 | 1 | 0,45 |
| (Larionow, Mackiewicz, et al., 2024) | heas_af | PHQ-2 | dep | 634 | 28,12 | 10,73 | 81,39 | 0 | 1 | 0,33 |
|  | heas_af | PHQ-2 | anx | 634 | 28,12 | 10,73 | 81,39 | 0 | 1 | 0,34 |
|  | heas_af | WHO-5 | wb | 634 | 28,12 | 10,73 | 81,39 | 0 | 1 | -0,25 |
| (Mathé et al., 2023) | heas_af | DASS-21 | anx | 275 | 31,16 | 13,87 | 66,18 | 0 | 1 | 0,34 |
|  | heas_af | DASS-21 | dep | 275 | 31,16 | 13,87 | 66,18 | 0 | 1 | 0,42 |
|  | heas_af | DASS-21 | stress | 275 | 31,16 | 13,87 | 66,18 | 0 | 1 | 0,46 |
|  | heas_af | STAI-Y | anx | 275 | 31,16 | 13,87 | 66,18 | 0 | 1 | 0,48 |
| (Rohn et al., 2025) | heas_af | DASS-21 | anx | 256 | 34,2 | 13,83 | 69,53 | 0 | 1 | 0,38 |
|  | heas_af | DASS-21 | dep | 256 | 34,2 | 13,83 | 69,53 | 0 | 1 | 0,39 |
|  | heas_af | DASS-21 | stress | 256 | 34,2 | 13,83 | 69,53 | 0 | 1 | 0,4 |
| (Türkarslan et al., 2023) | heas_af | DASS-21 | anx | 605 | 26,54 | 8,25 | 69,92 | 0 | 1 | 0,37 |
|  | heas_af | DASS-21 | dep | 605 | 26,54 | 8,25 | 69,92 | 0 | 1 | 0,27 |
|  | heas_af | DASS-21 | stress | 605 | 26,54 | 8,25 | 69,92 | 0 | 1 | 0,32 |
| (Ali et al., 2024) | heas_anx | DASS-8 | dep | 829 | 22,24 | 3,76 | 84,6 | 0,33 | 1 | 0,27 |
|  | heas_anx | DASS-8 | anx | 829 | 22,24 | 3,76 | 84,6 | 0,33 | 1 | 0,28 |
|  | heas_anx | DASS-8 | stress | 829 | 22,24 | 3,76 | 84,6 | 0,33 | 1 | 0,29 |
| (Çimsir et al., 2024) | heas_anx | BSI | dep | 385 | 31,14 | 11,58 | 61,3 | 0,33 | 1 | 0,37 |
| (Er et al., 2024) | heas_anx | DASS-21 | anx | 609 | - | - | 84,2 | 0,33 | 1 | 0,424 |
|  | heas_anx | DASS-21 | dep | 609 | - | - | 84,2 | 0,33 | 1 | 0,431 |
|  | heas_anx | DASS-21 | stress | 609 | - | - | 84,2 | 0,33 | 1 | 0,431 |
| (Heinzel et al., 2023) | heas_anx | DASS-21 | anx | 486 | 29,43 | 10,63 | 73,5 | 0,33 | 1 | 0,28 |
|  | heas_anx | DASS-21 | dep | 486 | 29,43 | 10,63 | 73,5 | 0,33 | 1 | 0,28 |
|  | heas_anx | DASS-21 | stress | 486 | 29,43 | 10,63 | 73,5 | 0,33 | 1 | 0,29 |
| (T. L. Hogg et al., 2021) | heas_anx | DASS-21 | anx | 365 | 19,90 | 3,59 | 79,7 | 0,33 | 1 | 0,28 |
|  | heas_anx | DASS-21 | dep | 365 | 19,90 | 3,59 | 79,7 | 0,33 | 1 | 0,21 |
|  | heas_anx | DASS-21 | stress | 365 | 19,90 | 3,59 | 79,7 | 0,33 | 1 | 0,27 |
| (T. L. Hogg et al., 2024) | heas_anx | GAD-7 | anx | 530 | 39,49 | 16,46 | 63,2 | 0,33 | 1 | 0,44 |
|  | heas_anx | DASS-21 | dep | 530 | 39,49 | 16,46 | 63,2 | 0,33 | 1 | 0,37 |
|  | heas_anx | SLS | wb | 530 | 39,49 | 16,46 | 63,2 | 0,33 | 1 | -0,11 |
| (T. Hogg et al., 2024) | heas_anx | BPSWQ | anx | 501 | 41,89 | 12,08 | 50,1 | 0,33 | 1 | 0,33 |
|  | heas_anx | BPSWQ | anx | 508 | 36,25 | 12,90 | 49,8 | 0,33 | 1 | 0,38 |
| (Larionow, Mackiewicz, et al., 2024) | heas_anx | PHQ-2 | dep | 634 | 28,12 | 10,73 | 81,39 | 0,33 | 1 | 0,21 |
|  | heas_anx | PHQ-2 | anx | 634 | 28,12 | 10,73 | 81,39 | 0,33 | 1 | 0,22 |
|  | heas_anx | WHO-5 | wb | 634 | 28,12 | 10,73 | 81,39 | 0,33 | 1 | -0,15 |
| (Mathé et al., 2023) | heas_anx | DASS-21 | anx | 275 | 31,16 | 13,87 | 66,18 | 0,33 | 1 | 0,26 |
|  | heas_anx | DASS-21 | dep | 275 | 31,16 | 13,87 | 66,18 | 0,33 | 1 | 0,34 |
|  | heas_anx | DASS-21 | stress | 275 | 31,16 | 13,87 | 66,18 | 0,33 | 1 | 0,37 |
|  | heas_anx | STAI-Y | anx | 275 | 31,16 | 13,87 | 66,18 | 0,33 | 1 | 0,34 |
| (Rohn et al., 2025) | heas_anx | DASS-21 | anx | 256 | 34,2 | 13,83 | 69,53 | 0,33 | 1 | 0,25 |
|  | heas_anx | DASS-21 | dep | 256 | 34,2 | 13,83 | 69,53 | 0,33 | 1 | 0,32 |
|  | heas_anx | DASS-21 | stress | 256 | 34,2 | 13,83 | 69,53 | 0,33 | 1 | 0,35 |
| (Türkarslan et al., 2023) | heas_anx | DASS-21 | anx | 605 | 26,54 | 8,25 | 69,92 | 0,33 | 1 | 0,3 |
|  | heas_anx | DASS-21 | dep | 605 | 26,54 | 8,25 | 69,92 | 0,33 | 1 | 0,29 |
|  | heas_anx | DASS-21 | stress | 605 | 26,54 | 8,25 | 69,92 | 0,33 | 1 | 0,31 |
| (Ali et al., 2024) | heas_be | DASS-8 | dep | 829 | 22,24 | 3,76 | 84,6 | 1 | 0,67 | 0,35 |
|  | heas_be | DASS-8 | anx | 829 | 22,24 | 3,76 | 84,6 | 1 | 0,67 | 0,33 |
|  | heas_be | DASS-8 | stress | 829 | 22,24 | 3,76 | 84,6 | 1 | 0,67 | 0,43 |
| (Çimsir et al., 2024) | heas_be | BSI | dep | 385 | 31,14 | 11,58 | 61,3 | 1 | 0,67 | 0,43 |
| (Er et al., 2024) | heas_be | DASS-21 | anx | 609 | - | - | 84,2 | 1 | 0,67 | 0,495 |
|  | heas_be | DASS-21 | dep | 609 | - | - | 84,2 | 1 | 0,67 | 0,463 |
|  | heas_be | DASS-21 | stress | 609 | - | - | 84,2 | 1 | 0,67 | 0,473 |
| (Heinzel et al., 2023) | heas_be | DASS-21 | anx | 486 | 29,43 | 10,63 | 73,5 | 1 | 0,67 | 0,4 |
|  | heas_be | DASS-21 | dep | 486 | 29,43 | 10,63 | 73,5 | 1 | 0,67 | 0,49 |
|  | heas_be | DASS-21 | stress | 486 | 29,43 | 10,63 | 73,5 | 1 | 0,67 | 0,42 |
| (T. L. Hogg et al., 2021) | heas_be | DASS-21 | anx | 365 | 19,90 | 3,59 | 79,7 | 1 | 0,67 | 0,31 |
|  | heas_be | DASS-21 | dep | 365 | 19,90 | 3,59 | 79,7 | 1 | 0,67 | 0,35 |
|  | heas_be | DASS-21 | stress | 365 | 19,90 | 3,59 | 79,7 | 1 | 0,67 | 0,3 |
| (T. L. Hogg et al., 2024) | heas_be | GAD-7 | anx | 530 | 39,49 | 16,46 | 63,2 | 1 | 0,67 | 0,61 |
|  | heas_be | DASS-21 | dep | 530 | 39,49 | 16,46 | 63,2 | 1 | 0,67 | 0,54 |
|  | heas_be | SLS | wb | 530 | 39,49 | 16,46 | 63,2 | 1 | 0,67 | -0,25 |
| (T. Hogg et al., 2024) | heas_be | BPSWQ | anx | 501 | 41,89 | 12,08 | 50,1 | 1 | 0,67 | 0,21 |
|  | heas_be | BPSWQ | anx | 508 | 36,25 | 12,90 | 49,8 | 1 | 0,67 | 0,25 |
| (Larionow, Mackiewicz, et al., 2024) | heas_be | PHQ-2 | dep | 634 | 28,12 | 10,73 | 81,39 | 1 | 0,67 | 0,33 |
|  | heas_be | PHQ-2 | anx | 634 | 28,12 | 10,73 | 81,39 | 1 | 0,67 | 0,25 |
|  | heas_be | SLS | wb | 634 | 28,12 | 10,73 | 81,39 | 1 | 0,67 | -0,28 |
| (Mathé et al., 2023) | heas_be | DASS-21 | anx | 275 | 31,16 | 13,87 | 66,18 | 1 | 0,67 | 0,37 |
|  | heas_be | DASS-21 | dep | 275 | 31,16 | 13,87 | 66,18 | 1 | 0,67 | 0,38 |
|  | heas_be | DASS-21 | stress | 275 | 31,16 | 13,87 | 66,18 | 1 | 0,67 | 0,41 |
|  | heas_be | STAI-Y | anx | 275 | 31,16 | 13,87 | 66,18 | 1 | 0,67 | 0,33 |
| (Rohn et al., 2025) | heas_be | DASS-21 | anx | 256 | 34,2 | 13,83 | 69,53 | 1 | 0,67 | 0,36 |
|  | heas_be | DASS-21 | dep | 256 | 34,2 | 13,83 | 69,53 | 1 | 0,67 | 0,42 |
|  | heas_be | DASS-21 | stress | 256 | 34,2 | 13,83 | 69,53 | 1 | 0,67 | 0,38 |
| (Türkarslan et al., 2023) | heas_be | DASS-21 | anx | 605 | 26,54 | 8,25 | 69,92 | 1 | 0,67 | 0,34 |
|  | heas_be | DASS-21 | dep | 605 | 26,54 | 8,25 | 69,92 | 1 | 0,67 | 0,27 |
|  | heas_be | DASS-21 | stress | 605 | 26,54 | 8,25 | 69,92 | 1 | 0,67 | 0,29 |
| (Ali et al., 2024) | heas_rum | DASS-8 | dep | 829 | 22,24 | 3,76 | 84,6 | 0,67 | 0,33 | 0,17 |
|  | heas_rum | DASS-8 | anx | 829 | 22,24 | 3,76 | 84,6 | 0,67 | 0,33 | 0,18 |
|  | heas_rum | DASS-8 | stress | 829 | 22,24 | 3,76 | 84,6 | 0,67 | 0,33 | 0,2 |
| (Çimsir et al., 2024) | heas_rum | BSI | dep | 385 | 31,14 | 11,58 | 61,3 | 0,67 | 0,33 | 0,2 |
| (Er et al., 2024) | heas_rum | DASS-21 | anx | 609 | - | - | 84,2 | 0,67 | 0,33 | 0,388 |
|  | heas_rum | DASS-21 | dep | 609 | - | - | 84,2 | 0,67 | 0,33 | 0,399 |
|  | heas_rum | DASS-21 | stress | 609 | - | - | 84,2 | 0,67 | 0,33 | 0,424 |
| (Heinzel et al., 2023) | heas_rum | DASS-21 | anx | 486 | 29,43 | 10,63 | 73,5 | 0,67 | 0,33 | 0,23 |
|  | heas_rum | DASS-21 | dep | 486 | 29,43 | 10,63 | 73,5 | 0,67 | 0,33 | 0,27 |
|  | heas_rum | DASS-21 | stress | 486 | 29,43 | 10,63 | 73,5 | 0,67 | 0,33 | 0,23 |
| (T. L. Hogg et al., 2021) | heas_rum | DASS-21 | anx | 365 | 19,90 | 3,59 | 79,7 | 0,67 | 0,33 | 0,22 |
|  | heas_rum | DASS-21 | dep | 365 | 19,90 | 3,59 | 79,7 | 0,67 | 0,33 | 0,15 |
|  | heas_rum | DASS-21 | stress | 365 | 19,90 | 3,59 | 79,7 | 0,67 | 0,33 | 0,22 |
| (T. L. Hogg et al., 2024) | heas_rum | GAD-7 | anx | 530 | 39,49 | 16,46 | 63,2 | 0,67 | 0,33 | 0,46 |
|  | heas_rum | DASS-21 | dep | 530 | 39,49 | 16,46 | 63,2 | 0,67 | 0,33 | 0,36 |
|  | heas_rum | SLS | wb | 530 | 39,49 | 16,46 | 63,2 | 0,67 | 0,33 | -0,18 |
| (T. Hogg et al., 2024) | heas_rum | BPSWQ | anx | 501 | 41,89 | 12,08 | 50,1 | 0,67 | 0,33 | 0,21 |
|  | heas_rum | BPSWQ | anx | 508 | 36,25 | 12,90 | 49,8 | 0,67 | 0,33 | 0,3 |
| (Larionow, Mackiewicz, et al., 2024) | heas_rum | PHQ-2 | dep | 634 | 28,12 | 10,73 | 81,39 | 0,67 | 0,33 | 0,15 |
|  | heas_rum | PHQ-2 | anx | 634 | 28,12 | 10,73 | 81,39 | 0,67 | 0,33 | 0,14 |
|  | heas_rum | WHO-5 | wb | 634 | 28,12 | 10,73 | 81,39 | 0,67 | 0,33 | -0,09 |
| (Mathé et al., 2023) | heas_rum | DASS-21 | anx | 275 | 31,16 | 13,87 | 66,18 | 0,67 | 0,33 | 0,28 |
|  | heas_rum | DASS-21 | dep | 275 | 31,16 | 13,87 | 66,18 | 0,67 | 0,33 | 0,26 |
|  | heas_rum | DASS-21 | stress | 275 | 31,16 | 13,87 | 66,18 | 0,67 | 0,33 | 0,32 |
|  | heas_rum | STAI-Y | anx | 275 | 31,16 | 13,87 | 66,18 | 0,67 | 0,33 | 0,29 |
| (Rohn et al., 2025) | heas_rum | DASS-21 | anx | 256 | 34,2 | 13,83 | 69,53 | 0,67 | 0,33 | 0,22 |
|  | heas_rum | DASS-21 | dep | 256 | 34,2 | 13,83 | 69,53 | 0,67 | 0,33 | 0,26 |
|  | heas_rum | DASS-21 | stress | 256 | 34,2 | 13,83 | 69,53 | 0,67 | 0,33 | 0,27 |
| (Türkarslan et al., 2023) | heas_rum | DASS-21 | anx | 605 | 26,54 | 8,25 | 69,92 | 0,67 | 0,33 | 0,29 |
|  | heas_rum | DASS-21 | dep | 605 | 26,54 | 8,25 | 69,92 | 0,67 | 0,33 | 0,24 |
|  | heas_rum | DASS-21 | stress | 605 | 26,54 | 8,25 | 69,92 | 0,67 | 0,33 | 0,28 |
| (Çimsir et al., 2024) | heas_tot | BSI | anx | 385 | 31,14 | 11,58 | 61,3 | 0,31 | 0,67 | 0,47 |
|  | heas_tot | BSI | dep | 445 | 31,14 | 11,58 | 61,3 | 0,31 | 0,67 | 0,49 |
| (Mathers-Jones & Todd, 2023) | heas_tot | DASS-21 | anx | 96 | 20,86 | 3,44 | 70,8 | 0,31 | 0,67 | 0,383 |
|  | heas_tot | DASS-21 | dep | 96 | 20,86 | 3,44 | 70,8 | 0,31 | 0,67 | 0,359 |
|  | heas_tot | DASS-21 | stress | 96 | 20,86 | 3,44 | 70,8 | 0,31 | 0,67 | 0,271 |
| (Rohn et al., 2025) | heas_tot | DASS-21 | anx | 256 | 34,2 | 13,83 | 69,53 | 0,31 | 0,67 | 0,36 |
|  | heas_tot | DASS-21 | dep | 256 | 34,2 | 13,83 | 69,53 | 0,31 | 0,67 | 0,42 |
|  | heas_tot | DASS-21 | stress | 256 | 34,2 | 13,83 | 69,53 | 0,31 | 0,67 | 0,43 |

*Abbreviations:* ***cor*** *= correlation of eco-distress questionnaire and mental health impairments questionnaire;* ***n*** *= number of participants in study;* ***Psych_Cat*** *= Category of Mental Health Impairment with dep = depression, anx = anxiety, stress = stress, and wb = well-being;* ***Scale_Eco*** *= Eco-Distress Questionnaire;* ***Scale_Psych*** *= Questionnaire used to assess mental health impairments;* ***% anx*** *= percentage content overlap with ICD-11 definition of generalized anxiety disorder;* ***% dep*** *= percentage of content overlap with ICD-11 definition of depressive disorder*

*Abbreviations Questionnaires:* ***BDI-II*** *= Beck’s Depression Inventory;* ***BPSWQ*** *= Brief Penn State Worry Questionnaire;* ***BSI*** *= Brief Symptom Inventory;* ***CES-D 10*** *= 10-item Center for Epidemiologic Studies Depression Scale;* ***CWS*** *= Cambridge Worry Scale;* ***DASS-8*** *= Depression, Anxiety and Stress Scale-8 items;* ***DASS-21*** *= Depression Anxiety Stress Scale;* ***GAD-7*** *= Generalized Anxiety Disorder Scale;* ***HADS*** *= Hospital Anxiety and Depression scale;* ***K-6*** *= Kessler Generalized Psychological Distress Scale;* ***K-10 =*** *Kessler Distress Scale;* ***MHC-SD*** *= Mental Health Continuum Short Form;* ***MHI-38*** *= Mental Health Inventory;* ***PHQ-4*** *= Patient Health Questionnaire – 4;* ***PHQ-8*** *= Patient Health Questionnaire – 8;* ***PHQ-9*** *= Patient Health Questionnaire – 9;* ***PSWQ*** *= Penn State Worry Questionnaire;* ***SLS*** *= Satisfaction with Life Scale;* ***STAI-Y*** *= Spielberger Trait Anxiety Scale;* ***WEMWBS*** *= Warwick-Edinburgh Mental Well-Being Scale;* ***WHOQOL-BREF*** *= WHO Quality of Life Short Version;* ***WHO-5*** *= World health organization well-being index*

**Literature (n = 51)**

Ali, M., Jahan, A. M., & Enaas, A. (2024). Investigating the impact of climate change on mental health among libyan arabs: A validation study of the hogg eco-anxiety scale. *Current Psychology: A Journal for Diverse Perspectives on Diverse Psychological Issues*, *null*(null), null-null.

Asgarizadeh, Z., Gifford, R., & Colborne, L. (2023). Predicting climate change anxiety. *Journal of Environmental Psychology*, *90*, 1–10. psyh. https://doi.org/10.1016/j.jenvp.2023.102087

Beckord, J., Krakowczyk, J. B., Gebhardt, N., Geiser, L. S., Kamler, K., Nikendei, C., Skoda, E.-M., Teufel, M., & Bäuerle, A. (2024). Development and Validation of a Climate Change Version of the Man-Made Disaster-Related Distress Scale (CC-MMDS). *The Journal of Climate Change and Health*, 100356. https://doi.org/10.1016/j.joclim.2024.100356

Cameron, E. C., & Kagee, A. (2025). Psychological, Experiential, and Behavioral Predictors of Climate Change Anxiety Among South African University Students. *Trends in Psychology*. https://doi.org/10.1007/s43076-025-00444-0

Chan, H.-W., Tam, K.-P., & Clayton, S. (2024). Testing an integrated model of climate change anxiety. *Journal of Environmental Psychology*, *97*, 102368. https://doi.org/10.1016/j.jenvp.2024.102368

Çimsir, E., Sahin, M., & Akdogan, R. (2024). Unveiling the relationships between eco-anxiety, psychological symptoms and anthropocentric narcissism: The psychometric properties of the Turkish version of the Hogg eco-anxiety scale. *CAMBRIDGE PRISMS-GLOBAL MENTAL HEALTH*, *11*. https://doi.org/10.1017/gmh.2024.20

Clayton, S., & Karazsia, B. T. (2020). Development and validation of a measure of climate change anxiety. *Journal of Environmental Psychology*, *69*. psyh. https://doi.org/10.1016/j.jenvp.2020.101434

Coates, Z., Kelly, M., & Brown, S. (2024). The Relationship between Climate Anxiety and Pro-Environment Behaviours. *SUSTAINABILITY*, *16*(12). https://doi.org/10.3390/su16125211

Er, S., Murat, M., Ata, E., Kose, S., & Buzlu, S. (2024). Nursing students’ mental health: How does eco-anxiety effect? *INTERNATIONAL JOURNAL OF MENTAL HEALTH NURSING*, *33*(5), 1315–1326. https://doi.org/10.1111/inm.13320

Feather, G., & Williams, M. (2022a). A psychometric evaluation of the Climate Change Anxiety Scale. *New Zealand Journal of Psychology*.

Feather, G., & Williams, M. (2022b). The moderating effects of psychological flexibility and psychological inflexibility on the relationship between climate concern and climate-related distress. *Journal of Contextual Behavioral Science*, *23*, 137–143. psyh. https://doi.org/10.1016/j.jcbs.2021.12.007

Fekih-Romdhane, F., Malaeb, D., Postigo, A., Sakr, F., Dabbous, M., Khatib, S. E., Obeid, S., & Hallit, S. (2024). The relationship between climate change anxiety and psychotic experiences is mediated by death anxiety. *International Journal of Social Psychiatry*, *70*(3), 574–581. https://doi.org/10.1177/00207640231221102

Gebhardt, N., Schwaab, L., Friederich, H.-C., & Nikendei, C. (2023). The relationship of climate change awareness and psychopathology in persons with pre-existing mental health diagnoses. *Frontiers in Psychiatry*, *14*, 1274523. https://doi.org/10.3389/fpsyt.2023.1274523

Hajek, A., & König, H.-H. (2023). Climate Anxiety and Mental Health in Germany. *Climate*, *11*(8). https://doi.org/10.3390/cli11080158

Heinzel, S., Tschorn, M., Schulte-Hutner, M., Schäfer, F., Reese, G., Pohle, C., Peter, F., Neuber, M., Liu, S., Keller, J., Eichinger, M., & Bechtoldt, M. (2023). Anxiety in response to the climate and environmental crises: Validation of the Hogg Eco-Anxiety Scale in Germany. *Frontiers in Psychology*, *14*. https://doi.org/10.3389/fpsyg.2023.1239425

Hepp, J., Klein, S. A., Horsten, L. K., Urbild, J., & Lane, S. P. (2023). Introduction and behavioral validation of the climate change distress and impairment scale. *Scientific Reports*, *13*(1). https://doi.org/10.1038/s41598-023-37573-4

Hogg, T. L., Stanley, S. K., O’Brien, L. V., Watsford, C. R., & Walker, I. (2024). Clarifying the nature of the association between eco-anxiety, wellbeing and pro-environmental behaviour. *Journal of Environmental Psychology*, *95*, 102249. https://doi.org/10.1016/j.jenvp.2024.102249

Hogg, T. L., Stanley, S. K., O’Brien, L., V., Wilson, M. S., & Watsford, C. R. (2021). The Hogg Eco-Anxiety Scale: Development and validation of a multidimensional scale. *GLOBAL ENVIRONMENTAL CHANGE-HUMAN AND POLICY DIMENSIONS*, *71*. https://doi.org/10.1016/j.gloenvcha.2021.102391

Hogg, T., Stanley, S., & O’Brien, L. (2024). Validation of the Hogg Climate Anxiety Scale. *CLIMATIC CHANGE*, *177*(6). https://doi.org/10.1007/s10584-024-03726-1

Hussein, M. F., Osman, S. R., Abd El Megied, N., Goda, S. M., & Sayed Hassan, S. M. (2023). Relation between Climate Changes, Quality of Life and Psychological Status among Assiut Population: Online Based Survey. *Assiut Scientific Nursing Journal*, *11*(40), 364–373. https://doi.org/10.21608/asnj.2024.242940.1695

Innocenti, M., Santarelli, G., Faggi, V., Ciabini, L., Castellini, G., Galassi, F., & Ricca, V. (2022). Psychometric properties of the Italian version of the climate change worry scale. *The Journal of Climate Change and Health*, *6*, 100140. https://doi.org/10.1016/j.joclim.2022.100140

Larionow, P., Gawrych, M., Mackiewicz, J., Michalak, M., Mudło-Głagolska, K., Preece, D. A., & Stewart, A. E. (2024). The Climate Change Worry Scale (CCWS) and Its Links with Demographics and Mental Health Outcomes in a Polish Sample. *Healthcare (Basel, Switzerland)*, *12*(11). https://doi.org/10.3390/healthcare12111128

Larionow, P., Mackiewicz, J., Mudło-Głagolska, K., Michalak, M., Mazur, M., Gawrych, M., Komorowska, K., & Preece, D. A. (2024). Measuring Eco-Anxiety with the Polish Version of the 13-Item Hogg Eco-Anxiety Scale (HEAS-13): Latent Structure, Correlates, and Psychometric Performance. *Healthcare (Basel, Switzerland)*, *12*(22). https://doi.org/10.3390/healthcare12222255

Larionow, P., Sołtys, M., Izdebski, P., Mudło-Głagolska, K., Golonka, J., Demski, M., & Rosińska, M. (2022). Climate Change Anxiety Assessment: The Psychometric Properties of the Polish Version of the Climate Anxiety Scale. *Frontiers in Psychology*, *13*, 870392. https://doi.org/10.3389/fpsyg.2022.870392

Latkin, C., Dayton, L., Scherkoske, M., Countess, K., & Thrul, J. (2022). What predicts climate change activism?: An examination of how depressive symptoms, climate change distress, and social norms are associated with climate change activism. *The Journal of Climate Change and Health*, *8*, 100146. https://doi.org/10.1016/j.joclim.2022.100146

Lau, S. S., Appiah, K., Ho, C. C., Cheng, M. C., & Yang, B.-Y. (2025). Measuring Chinese negative emotion towards climate change: Psychometric properties of the Chinese version of Climate Change Anxiety Scale. *The Journal of Climate Change and Health*, *21*, 100364. https://doi.org/10.1016/j.joclim.2024.100364

Lukacs, J. N., Bratu, A., Adams, S., Logie, C., Tok, N., McCunn, L. J., Lem, M., Henley, A., Closson, K., Martin, G., Gislason, M. K., Takaro, T., & Card, K. G. (2023). The concerned steward effect: Exploring the relationship between climate anxiety, psychological distress, and self-reported climate related behavioural engagement. *Journal of Environmental Psychology*, *90*, 1–9. psyh. https://doi.org/10.1016/j.jenvp.2023.102091

Lutz, P. K., Passmore, H.-A., Howell, A. J., Zelenski, J. M., Ying Yang, & Richardson, M. (2023). The Continuum of Eco-Anxiety Responses: A Preliminary Investigation of Its Nomological Network. *Collabra: Psychology*, *9*(1), Article 1. a9h. https://doi.org/10.1525/collabra.67838

Lykins, A. D., Bonich, M., Sundaraja, C., & Cosh, S. (2024). Climate change anxiety positively predicts antenatal distress in expectant female parents. *Journal of Anxiety Disorders*, *101*, 102801. https://doi.org/10.1016/j.janxdis.2023.102801

Mathé, M., Grisetto, F., Gauvrit, N., & Roger, C. (2023). Psychometric validation of the French version of the Hogg Eco-Anxiety Scale (HEAS-FR). *Canadian Journal of Behavioural Science / Revue Canadienne Des Sciences Du Comportement*. psyh. https://doi.org/10.1037/cbs0000398

Mathers-Jones, J., & Todd, J. (2023). Ecological anxiety and pro-environmental behaviour: The role of attention. *Journal of Anxiety Disorders*, *98*, 102745. https://doi.org/10.1016/j.janxdis.2023.102745

Micoulaud-Franchi, J., Coelho, J., Geoffroy, P., Vecchierini, M., Poirot, I., Royant-Parola, S., Hartley, S., Cugy, D., Gronfier, C., Gauld, C., & Rey, M. (2024). Eco-anxiety: An adaptive behavior or a mental disorder? Results of a psychometric study. *ENCEPHALE-REVUE DE PSYCHIATRIE CLINIQUE BIOLOGIQUE ET THERAPEUTIQUE*, *50*(4), 406–415. https://doi.org/10.1016/j.encep.2023.08.009

Mouguiama-Daouda, C., Blanchard, M. A., Coussement, C., & Heeren, A. (2022). On the Measurement of Climate Change Anxiety: French Validation of the Climate Anxiety Scale. *Psychologica Belgica*, *62*(1), Article 1. https://doi.org/10.5334/pb.1137

Parmentier, M.-L., Weiss, K., Aroua, A., Betry, C., Rivière, M., & Navarro, O. (2024). The influence of environmental crisis perception and trait anxiety on the level of eco-worry and climate anxiety. *Journal of Anxiety Disorders*, *101*, 102799. https://doi.org/10.1016/j.janxdis.2023.102799

Plohl, N., Izidor Mlakar, Bojan Musil, & Urška Smrke. (2023). Measuring young individuals’ responses to climate change: Validation of the Slovenian versions of the Climate Anxiety Scale and the Climate Change Worry Scale. *PsyArXiv*. https://doi.org/10.31234/osf.io/kxzcn

Reyes, M. E. S., Carmen, B. P. B., Luminarias, M. E. P., Mangulabnan, S. A. N. B., & Ogunbode, C. A. (2023). An investigation into the relationship between climate change anxiety and mental health among Gen Z Filipinos. *Current Psychology (New Brunswick, N.J.)*, *42*(9), Article 9. https://doi.org/10.1007/s12144-021-02099-3

Rohn, N., Barke, A., & Forkmann, T. (2025). Wie krank macht der Klimawandel? Validierung einer deutschen Version der Hogg Eco-Anxiety Scale und ihr Zusammenhang mit Maßen psychischer Gesundheit. *VERHALTENSTHERAPIE*. https://doi.org/10.1159/000541505

Schwartz, S. E. O., Benoit, L., Clayton, S., Parnes, M. F., Swenson, L., & Lowe, S. R. (2022). Climate change anxiety and mental health: Environmental activism as buffer. *Current Psychology*. https://doi.org/10.1007/s12144-022-02735-6

Searle, K., & Gow, K. (2010). Do concerns about climate change lead to distress? *International Journal of Climate Change Strategies and Management*, *2*(4), Article 4. https://doi.org/10.1108/17568691011089891

Shareeff, N., Pramono, R. K., & Saliha, A. (2024). Climate Change and Mental Health: Exploring the Psychological Impacts of Environmental Distress in Indonesia. *Scientia Psychiatrica*, *5*(4), Article 4. https://doi.org/10.37275/scipsy.v5i4.179

Shepherd, S., Raynal, P., & Guedj, M. (2024). Psychometric properties of the French version of the climate change worry scale. *The Journal of Climate Change and Health*, *20*, 100361. https://doi.org/10.1016/j.joclim.2024.100361

Söder, A., Herr, R. M., Görig, T., & Diehl, K. (2025). Climate Change Worry in German University Students: Determinants and Associations with Health-Related Outcomes. *Climate*, *13*(2), Article 2. https://doi.org/10.3390/cli13020027

Stanley, S. K. (2023). Anticipatory solastalgia in the anthropocene: Climate change as a source of future-oriented distress about environmental change. *Journal of Environmental Psychology*, *91*, 1–5. psyh. https://doi.org/10.1016/j.jenvp.2023.102134

Stewart, A. E. (2021). Psychometric Properties of the Climate Change Worry Scale. *International Journal of Environmental Research and Public Health*, *18*(2), Article 2. https://doi.org/10.3390/ijerph18020494

Sümen, A., Kublay, Y., & Adibelli, D. (2025). Evaluation of Adult Individuals’ Climate Change Concern and Mental Well-Being Levels in Türkiye: A Descriptive and Correlational Study. *Public Health Nursing (Boston, Mass.)*, *42*(2), 655–664. https://doi.org/10.1111/phn.13493

Thomson, E. E., & Roach, S. P. (2023). The relationships among nature connectedness, climate anxiety, climate action, climate knowledge, and mental health. *Frontiers in Psychology*, *14*, 1241400. https://doi.org/10.3389/fpsyg.2023.1241400

Türkarslan, K. K., Kozak, E. D., & Yıldırım, J. C. (2023). Psychometric properties of the Hogg Eco-Anxiety Scale (HEAS-13) and the prediction of pro-environmental behavior. *Journal of Environmental Psychology*, *92*, 1–8. psyh. https://doi.org/10.1016/j.jenvp.2023.102147

Wagner, L., & Witthöft, M. (2024). Climate Anxiety-Appropriate or Dysfunctional? An Investigation of the Relationship With Depressiveness, Anxiety, and Pro-Environmen-tal Behavior. *ZEITSCHRIFT FUR KLINISCHE PSYCHOLOGIE UND PSYCHOTHERAPIE*, *53*(2), 59–71. https://doi.org/10.1026/1616-3443/a000754

Whitmarsh, L., Player, L., Jiongco, A., James, M., Williams, M., Marks, E., & Kennedy-Williams, P. (2022). Climate anxiety: What predicts it and how is it related to climate action? *Journal of Environmental Psychology*, *83*, 101866. https://doi.org/10.1016/j.jenvp.2022.101866

Wullenkord, M. C., Tröger, J., Hamann, K. R. S., Loy, L. S., & Reese, G. (2021). Anxiety and climate change: A validation of the Climate Anxiety Scale in a German-speaking quota sample and an investigation of psychological correlates. *Climatic Change*, *168*(3–4), Article 3–4. https://doi.org/10.1007/s10584-021-03234-6

Zeier, P., & Wessa, M. (2024). Measuring eco-emotions: A German version of questionnaires on eco-guilt, ecological grief, and eco-anxiety. *Discover Sustainability*, *5*(1), 29. https://doi.org/10.1007/s43621-024-00209-2
